# Targeting Dermal Fibroblast Subtypes in Antifibrotic Therapy: Surface Marker as a Cellular Identity or a Functional Entity?

**DOI:** 10.3389/fphys.2021.694605

**Published:** 2021-07-15

**Authors:** Xin Huang, Yimin Khoong, Chengyao Han, Dai Su, Hao Ma, Shuchen Gu, Qingfeng Li, Tao Zan

**Affiliations:** Department of Plastic and Reconstructive Surgery, Shanghai Ninth People’s Hospital, School of Medicine, Shanghai Jiao Tong University, Shanghai, China

**Keywords:** fibroblast subtypes, fibroblast heterogeneity, fibrosis, surface marker, antifibrotic therapy

## Abstract

Fibroblasts are the chief effector cells in fibrotic diseases and have been discovered to be highly heterogeneous. Recently, fibroblast heterogeneity in human skin has been studied extensively and several surface markers for dermal fibroblast subtypes have been identified, holding promise for future antifibrotic therapies. However, it has yet to be confirmed whether surface markers should be looked upon as merely lineage landmarks or as functional entities of fibroblast subtypes, which may further complicate the interpretation of cellular function of these fibroblast subtypes. This review aims to provide an update on current evidence on fibroblast surface markers in fibrotic disorders of skin as well as of other organ systems. Specifically, studies where surface markers were treated as lineage markers and manipulated as functional membrane proteins are both evaluated in parallel, hoping to reveal the underlying mechanism behind the pathogenesis of tissue fibrosis contributed by various fibroblast subtypes from multiple angles, shedding lights on future translational researches.

## Introduction

Fibroblast (Fb), as a vital interstitial cell, is involved in a wide variety of biological functions such as conferring structural support to tissues, secreting extracellular matrix (ECM), participating in tissue damage repair and immune responses (Lynch and Watt, 2018). Besides, Fb is also the chief effector cell in fibrotic diseases. Abnormal Fb function, such as cellular hyperproliferation and excessive extracellular matrix deposition, can directly mediate tissue fibrosis (Jiang and Rinkevich, 2020). Fb has always been thought to be stable and unitary in terms of its composition and function. In the past, based on the histological characteristics of the skin, Fb was mainly classified into three types, namely papillary layer, deep reticular layer, hair shaft and papillary region of the hair follicle (Lynch and Watt, 2018). Unfortunately, due to the lack of surface markers that can effectively distinguish different fibroblast subtypes, accurate separation of different fibroblast subtypes has always been an obstacle, and has greatly restricted the further in-depth study of the functional characteristics of this complex cell population.

In recent years, development of omic sequencing technology, especially in single-cell omics, has enabled people to understand the phenotype and functional characteristics of cells on the single-cell level, thereby increased the depth and accuracy in our understanding of the complex cell populations (Prakadan et al., 2017). With the help of omics technology, fibroblast subpopulations were studied in detail and were found to be highly heterogeneous in their compositions. Previously characterized papillary and reticular Fb were demonstrated to contain a complex population of cells that do not share a specific marker (Ascensión et al., 2020; Vorstandlechner et al., 2020). At present, several studies have been performed to uncover the Fb heterogeneity in normal human skin tissues. Tabib et al. (2018) discovered that dermal Fb can be divided into two main subpopulations characterized by SFRP2 and FMO1; and into several subpopulations according to their expression of CRABP1, COL11A1, FMO2, PRG4, and C2ORF40. Philippeos et al. (2018) performed transcriptome sequencing on the papillary and reticular dermal tissues, and revealed several pan-Fb markers, including CD90, platelet-derived growth factor receptor (PDGFR, including PDGFRα and PDGFRβ). Besides, CD39, COL6A5, COL23A1, APCDD1, HSPB3, and WIF1 were found highly expressed in papillary Fb, while CD36 was specifically highly expressed in reticular Fb (Philippeos et al., 2018). Using flow cytometry, Korosec et al. (2019) found that Fbs characterized by fibroblast activation protein (FAP) positive and CD90 negative phenotype are enriched in the papillary dermis and expressed both PDPN and NTN1, displayed active proliferation, and are relatively resistant to adipogenic differentiation. On the other hand, FAP-CD90+ Fbs expressed high levels of ACTA2, MGP, PPARγ, and CD36 and possessed a higher adipogenic potential, contributing to features of reticular Fbs.

The research on Fb heterogeneity in healthy skin is still very much in its infancy. Based on currently available evidence, it is acknowledged that pan-Fb surface markers of human skin Fb are CD90, PDGFR α and PDGFRβ, while the surface markers of Fb subtypes are FAP, CD26, CD36, and CD39. These surface markers hold the promise of future antifibrotic therapies by targeting Fb subtypes with small molecule inhibitors or inhibitory antibodies. However, evidence regarding the functional characteristics and dynamic alteration of Fb subtypes in both healthy or fibrotic skin is still scarce. More importantly, there is a discrepancy between Fb surface markers as lineage markers or as functional entities in the previous studies, which further complicates the interpretation of cellular functions, hampering future development of targeted therapy (Jacob et al., 2012). In this review, we aim to summarize the current evidence of Fb surface markers in fibrotic disorders of skin as well as of other organ systems. Specifically, we would like to compare the role of these surface markers in fibrotic diseases as lineage markers or as functional membrane proteins. Besides, antifibrotic therapies targeting certain Fb subtypes or particular surface marker proteins would be evaluated, hoping to shed light on the significance of these Fb subtypes during the fibrotic process, and to provide some valuable insights for future translational research.

## Pan Fibroblast Surface Markers

### CD90

CD90, also known as Thy1, is a glycosylphosphatidylinositol-anchored glycoprotein that is expressed on the surfaces of T cells, neuronal cells, endothelial cells, mesenchymal stem cells and fibroblasts (Jiang and Rinkevich, 2018). CD90 regulates cell adhesion and migration, and plays an important role in the processes of axon growth, T cell activation, cell proliferation and apoptosis regulation, and tumor cell migration (Shaikh et al., 2016). In human dermis, it was shown that CD90 is widely expressed in all skin layers, including papillary dermis, reticular dermis and hypodermis, thus it is also known as a pan-Fb surface marker (Driskell et al., 2013; Philippeos et al., 2018). However, Korosec et al. (2019) stated that the uppermost papillary Fbs possessed a CD90 negative phenotype. Besides, CD90 cannot clearly distinguish between dermal mesenchymal stem cells and dermal Fbs, suggesting that CD90 alone is not an accurate marker to define Fbs in general or its subtypes (Jiang and Rinkevich, 2018).

CD90+ Fbs were found to be accumulated in the collagen packed loci of several fibrotic diseases, including systemic sclerosis (Nazari et al., 2016), cholestatic liver injury (Katsumata et al., 2017), pathological scarring (Ho et al., 2019) and contracted capsule induced by tissue expander implantation (Hansen et al., 2017). Moreover, the expression level of CD90 in Fbs was positively correlated with the severity of tissue fibrosis (Nazari et al., 2016; Katsumata et al., 2017), suggesting the positive role of CD90+ Fbs in the pathogenesis of these diseases. CD90+ Fbs were also regarded to be functionally activated as myofibroblast marker α-smooth muscle actin (αSMA) and ECM related genes were both highly expressed (Hansen et al., 2017; Ho et al., 2019). Besides, CD90+ Fbs inhibited ECM degradation through the upregulation of Tissue Inhibitors of Metalloproteinases-1 (TIMP-1) (Katsumata et al., 2017).

However, CD90 exerted contradictory functions in different disease models. For instance, the depletion of CD90 would halt fibrosis in prosthesis-induced scar formation (Hansen et al., 2017) and idiopathic pulmonary fibrosis (IPF) (Fiore et al., 2015). These antifibrotic effects may be attributed to the inhibition of the association between CD90 and the αvβ3 integrins upon CD90 depletion, further blocking Src family kinase recruitment and Rho signaling activation (Gerber et al., 2013; Fiore et al., 2015). In contrast, in IPF, CD90 was found to be lowly expressed in the fibroblastic foci (Sanders et al., 2008). Also, Lung Fbs from CD90 knockout mice showed increased cell proliferation and collagen deposition, revealing antifibrotic properties of CD90 (Nicola et al., 2009). The inconsistencies across studies suggested that: (1) CD90+ Fb may act differently during fibrosis formation depending on the organ system involved; (2) CD90 is widely expressed in multiple mesenchymal cells, thus global depletion of CD90 cells may not be limited to just CD90+ Fbs, but also other CD90+ cells, thereby significantly complicating the interpretation of results (Rege and Hagood, 2006).

**FIGURE 1:**
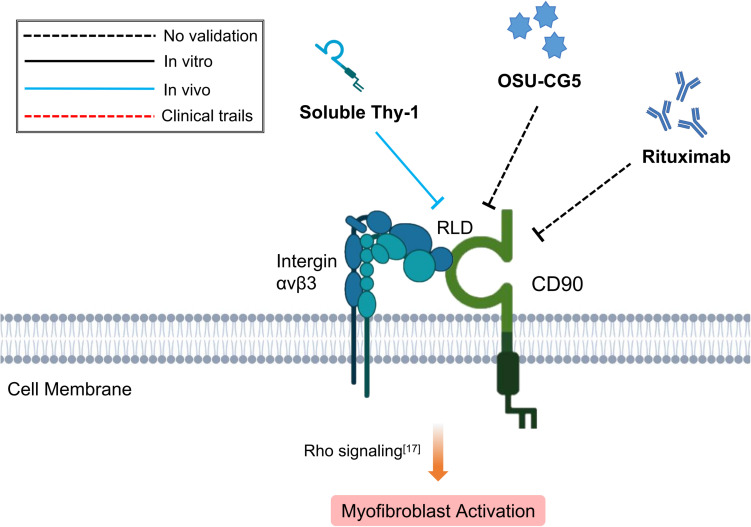
Schematic graph showing potential antifibrotic therapies targeting CD90. (RLD, RGD-like tripeptide).

Small molecular compounds such as OSU-CG5 and monoclonal antibodies targeting CD90 have already been used to inhibit CD90+ tumor cells in solid or hematological malignancy (Ishiura et al., 2010; Chen et al., 2015). However, to date none has been tested for treatment of fibrotic diseases. Apart from directly targeting CD90 *per se* or CD90+ cells, other treatment options which inhibits the differentiation of endothelial cells into CD90+ Fb (Wei et al., 2020) or disrupts the interaction between CD90 and integrin αν (Tan et al., 2019), might also achieve desirable therapeutic effects on tissue fibrosis (Figure 1).

### Platelet-Derived Growth Factor Receptor (PDGFR)

Platelet-derived growth factor receptor belongs to the receptor tyrosine-specific protein kinase family. It possesses intrinsic kinase activity and is widely expressed in Fbs, endothelial cells and myoepithelial cells (Lynch and Watt, 2018). The binding of PDGF isoforms to PDGFR dimers αα, αβ, ββ would trigger autophosphorylation of PDGFRs on different tyrosine residues and subsequent activation of downstream signaling pathways, regulating cell proliferation, apoptosis, differentiation, migration, and angiogenesis (Östman, 2017; Klinkhammer et al., 2018). It plays important roles in physiological processes including growth and development, and wound repair; as well as in pathological processes such as tumorigenesis (Pietras et al., 2003).

In normal human skin, PDGFRα and PDGFRβ are indiscriminately expressed in the papillary and reticular dermis (Philippeos et al., 2018). It has been reported that upon muscle and skin injury, a lineage of ADAM12+ cells would be induced into a distinct subset of PDGFRα+ cells, namely the ADAM12+ PDGFRα+ Fbs which mediates scarring repair by producing collagen (Dulauroy et al., 2012). Similar fibrogenic potential of PDGFRα+ cells has also been observed in other organs. It has been reported that PDGFRα+ progenitor cells give rise to major matrix-producing Fbs in tendon repair (Harvey et al., 2019), liver fibrosis (Ramachandran et al., 2019), and kidney and heart ischemic injury (Santini et al., 2020). PDGF-enriched microenvironment would also contribute to tissue fibrosis as seen in Duchenne muscular dystrophy (DMD), where PDGFRα + Sca1 + CD45− mesenchymal progenitor cells would be activated into tissue remodeling cells after receiving PDGF-AA ligands from the surrounding muscle cells (Ieronimakis et al., 2016). Besides, PDGFRα, which is also expressed in the adipose precursor cells (Driskell and Watt, 2015; Marcelin et al., 2017), would be activated, resulting in the transformation of cells into PDGFRα + CD9^high^ Fbs that act as the pivotal cells in tissue metabolism and white adipose tissue (WAT) fibrosis (Marcelin et al., 2017).

Profibrotic effect of PDGF signaling pathway has been evaluated in multiple organs including liver (Hayes et al., 2014; Ramachandran et al., 2019), skin (Olson and Soriano, 2009), kidney (Ostendorf et al., 2003) and heart (Pontén et al., 2003). Other than activating the classic fibrogenic ERK, AKT, and NF-κB pathways which ultimately resulting in excessive tissue fibrosis (Kocabayoglu et al., 2015; Higashi et al., 2017), PDGFR signaling, specifically PDGFβ signaling is also accountable for functional activation of Fbs as shown by upregulation of αSMA and profibrotic cytokines such as matrix metalloproteinases (MMPs) and TIMPs (Czochra et al., 2006). In addition, PDGF-BB is involved in promoting the secretion of extracellular vesicles containing PDGFRα, which in turn facilitates the activation of cellular function of hepatic stellate cells, promoting liver fibrosis (Kostallari et al., 2018).

As the critical role of PDGF/PDGFR signaling in promoting tissue fibrosis has been well documented, numerous antifibrotic approaches targeting this pathway have been developed (Papadopoulos et al., 2018). Basically, these treatment strategies are mainly divided into three categories (Papadopoulos et al., 2018): (1) sequestering PDGF ligands or inhibiting their binding to their respective receptors using neutralizing antibodies or aptamers, which are single-stranded DNA or RNA molecules that possess selective binding affinity to the PDGF ligands, consequently blocking the activation of PDGFRs; (2) inhibiting ligand-receptor interactions by blocking the extracellular domain of PDGFR with antibodies or small molecular drugs; (3) blocking the activation of intracellular tyrosine kinase or downstream pathways of PDGFR signaling with low molecular weight inhibitors.

**FIGURE 2:**
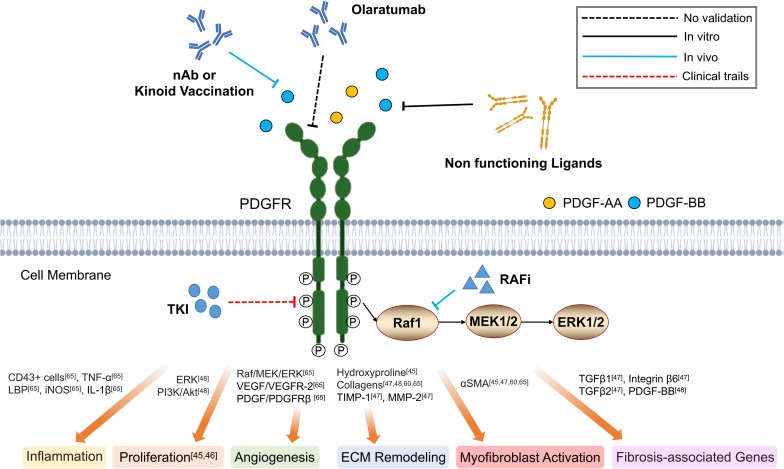
Schematic graph showing potential antifibrotic therapies targeting PDGFR. (nAb, neutralizing antibody; TKI, tyrosine kinase inhibitor; RAFi, Raf kinase inhibitor; LBP, lipopolysaccharide-binding protein; TNF-α, tumor necrosis factor-α; iNOS, inducible nitric oxide synthase; IL-1β, interleukin-1β; PI3K, phosphoinositide 3-kinase; Akt, also known as PKB, protein kinase B; VEGF, vascular endothelial growth factor; VEGFR-2, vascular endothelial growth factor receptor-2; TIMP-1, tissue inhibitors of metalloproteinases-1; MMP-2, matrix metalloproteinase-2; αSMA, α-smooth muscle actin; ECM, extracellular matrix).

Hao et al. (2012) demonstrated that PDGF-B kinoid immunogen, a kind of PDGF-B-derived epitope-carrier protein heterocomplexes, would elicit the production of neutralizing anti-PDGF-B autoantibodies responsible for the suppression of proliferation and activation of the hepatic stellate cells (HSCs), which would ultimately inhibit liver fibrosis. Similar antifibrotic effects can also be achieved through direct administration of PDGF-BB specific neutralizing antibody (MOR8457) (Yoshida et al., 2014; Kuai et al., 2015) or soluble dominant negative PDGFRβ (Borkham-Kamphorst et al., 2004), as demonstrated in mice model of hepatic fibrosis. For small molecular drugs, tyrosine kinase inhibitors (TKIs) have been proven to be one of the most promising antifibrotic therapies that target the enzymatic activity of PDGFR (Papadopoulos et al., 2018), such as in hepatic (Liu et al., 2011; Shaker et al., 2011) and pulmonary (Vuorinen et al., 2007; Fleetwood et al., 2017) fibrosis. With respect to skin fibrosis, Imatinib inhibited the proliferation and production of ECM, including collagen 1 and fibronectin *in vitro* in dermal Fbs obtained from systemic sclerosis patients (Distler et al., 2007; Soria et al., 2008). *In vivo*, Imatinib administration reduced dermal thickening and prevented the differentiation of resting Fbs into myofibroblasts in TSK-1 mice and bleomycin-induced dermal fibrosis (Akhmetshina et al., 2009). Similar protective effects on dermal fibrotic diseases would also be observed with Sunitinib, Dasatinib, and Nilotinib *in vivo* (Akhmetshina et al., 2008; Kavian et al., 2012). However, clinical trials evaluating the therapeutic efficacy of Imatinib on systemic sclerosis gave multifarious results (Khanna et al., 2011; Spiera et al., 2011; Prey et al., 2012; Fraticelli et al., 2014; Gordon et al., 2014). Kay and High (2008) first reported progressive improvement of skin thickening and tethering following initiation of Imatinib in two patients with nephrogenic systemic fibrosis. However, recurrence of skin lesions were observed shortly after therapy withdrawal (Kay and High, 2008). In 26 systemic sclerosis interstitial lung disease, the use of low dose Imatinib (200 mg/day) for 6 months was associated with good drug tolerance and stabilized lung function, however, without significant effects on skin lesions (Fraticelli et al., 2014). Another phase 2 trial showed that the use of Imatinib 400 mg/day would improve both forced vital capacity and skin thickening (Spiera et al., 2011; Gordon et al., 2014). Yet, using similar dose of Imatinib (400 mg/day), Prey et al. (2012) failed to demonstrate therapeutic efficacy of Imatinib in regards to impact on dermal thickness, pulmonary function and quality of life. By further increasing the dose to 600 mg/day, Khanna et al. (2011) only reported a trend toward improved lung function and skin thickness, but were associated with significant adverse effects. The reasons for the inconsistencies between these studies are unclear, but may be due to drug dosage, treatment duration, and patient groups and thus requires further investigation (Khanna et al., 2011). In conclusion, the curative effect of TKIs in the treatment of fibrotic disease requires further studies to confirm its effectiveness (Figure 2).

It has been reported that the auto-phosphorylated PDGFR and downstream activation of Ras, RAF pro-oncogene serine/threonine protein kinase (RAF-1), mitogen-activated protein kinase (MEK) and extracellular signal-regulated protein kinase (ERK) signaling pathways facilitate the progression of hepatic fibrosis (Ying et al., 2017). Sorafenib, a potent inhibitor of PDFGRβ and RAF kinase, has been demonstrated to effectively reduce the portal pressure and portosystemic collaterals in a rat model of portal hypertension, thereby reducing the level of intrahepatic fibrosis (Figure 2) (Mejias et al., 2009).

## Surface Markers for Fibroblast Subtypes

### Fibroblast Activation Protein (FAP)

Fibroblast activation protein is an integral membrane glycoprotein of the serine proteases family, possessing dual collagenase and dipeptidase activities, which aids in the degradation of gelatin, type I collagen and a variety of dipeptides (Kelly, 2005). Although FAP and CD26 both belong to the same S9B prolyl oligopeptidase subfamily and are highly homologous, they are not interchangeable (Kelly, 2005). Previous studies reported that FAP was highly expressed in the cancer associated fibroblasts (CAFs), which in turn mediated cancer invasion and metastasis through the degradation of extracellular matrix (Kalluri and Zeisberg, 2006).

In normal human skin, FAP + CD90- Fbs are commonly regarded as the papillary Fbs, which showed increased proliferation potential and lower adipogenic differentiation as compared to the reticular Fbs (Korosec et al., 2019). FAP is also found to be highly expressed in the collagen-accumulated loci of several fibrotic diseases including keloid (Dienus et al., 2010), liver fibrosis (Levy et al., 2002), myocardial infarction (Tillmanns et al., 2015), lung fibrosis (Acharya et al., 2006), Crohn’s disease (Truffi et al., 2018), and arthritis (Croft et al., 2019). FAP+ Fbs generally highly express αSMA as observed in infarcted heart tissues of human and mice model, suggesting these Fbs express an activated contractive phenotype (Tillmanns et al., 2015). Further, Avery et al. found that the expression of FAP and αSMA is regulated by ECM composition, elasticity and transforming growth factor-β (TGF-β) signaling (Avery et al., 2018). In fibronectin-enriched matrix, TGF-β preferentially upregulates the expression of FAP; whereas in Collagen 1-enriched matrix, αSMA is induced instead (Avery et al., 2018). FAP^Hi^αSMA^low^ and FAP^low^αSMA^Hi^ Fbs displayed distinct functional differences in that the former has a higher capacity of ECM deposition, while the latter showed a more contractive potential (Avery et al., 2018). In addition, FAP has been reported to be vital for various cellular functions like cell proliferation (Croft et al., 2019), migration (Wang et al., 2005; Dienus et al., 2010), invasion (Wang et al., 2005; Dienus et al., 2010), apoptosis (Wang et al., 2005) and production of profibrotic proteins (such as TIMPs) (Truffi et al., 2018). The therapeutic effects of FAP targeted approaches are partly attributed to the regulation of cellular functions of the culprit FAP expressing cells. However, it has been noted that the choice of animal model or intervention approach would influence the final interpretation of therapeutic effect by targeting the FAP+ Fbs (Kimura et al., 2019). For instance, FAP+ cell depletion by T cells expressing FAP chimeric antigen receptors or global FAP knockout showed increased level of pulmonary fibrosis in bleomycin-induced lung fibrosis, but had only minimal effects on the Ad-TGFβ induced model (Kimura et al., 2019). These phenomena highlight the fact that different fibrosis models may have distinct mechanisms of action upon initiating insults and that FAP+ Fbs may have adapted to different functions throughout the fibrotic processes.

**FIGURE 3:**
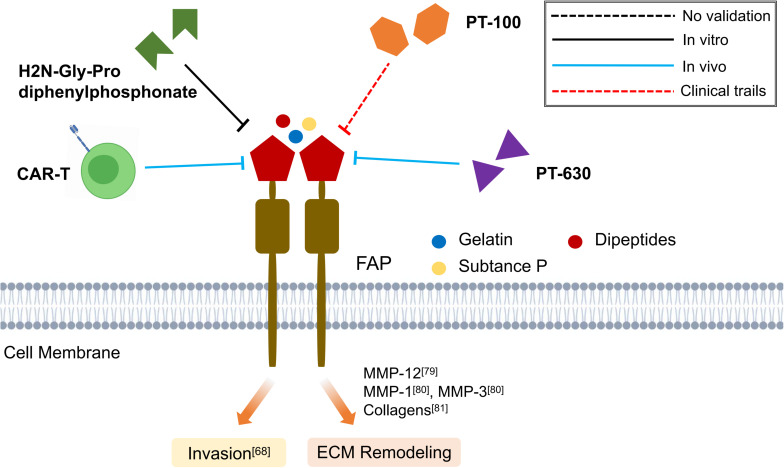
Schematic graph showing potential antifibrotic therapies targeting FAP. (CAR-T, chimeric antigen receptors engineered T cell; MMP, matrix metalloproteinase; ECM, extracellular matrix).

Although various FAP targeting strategies have been established, including inhibition of enzymatic activity of FAP, depletion of FAP expressing cells and targeted delivery of cytotoxic compounds, their applications in fibrotic diseases have been limited so far (Figure 3) (Busek et al., 2018).

Targeted inhibition of FAP using H2N-Gly-Pro diphenylphosphonate, an irreversible inhibitor of FAP (Gilmore et al., 2006), has been shown to impair the invasiveness of keloid Fbs *in vitro* (Dienus et al., 2010). Similarly, Talabostat mesylate (PT-100), an extracellular dipeptidylpeptidases inhibitor, would reduce bleomycin-induced lung injury by downregulating FAP and MMP-12 expression and increasing macrophage activation (Egger et al., 2017). Moreover, oral administration of L-glutamyl L-boroproline (PT-630), a more specific FAP inhibitor, in murine model of rheumatoid arthritis has found to exert inhibitory effects on the invasiveness of synovial Fbs (Ospelt et al., 2010). However, PT-630 which is relatively non-selective often inhibits both CD26 and FAP activities, thus the development of more selective FAP inhibitors is warranted, as well as the assessment of their antifibrotic activity in preclinical and clinical trials (Jacob et al., 2012). Recently, chimeric antigen receptors (CARs) engineered T cells that specifically target the FAP have been demonstrated to prevent cardiac fibrosis and improve cardiac function *in vivo*, suggesting a novel direction to develop anti-FAP therapies (Aghajanian et al., 2019) (Figure 3).

### CD26

CD26 (also known as dipeptidyl peptidase IV, DPP IV) is a highly conserved type II transmembrane serine exopeptidase that hydrolyzes proline or alanine from the N-terminus of a broad range of polypeptides (Hu and Longaker, 2016). CD26 is widely expressed in a variety of cells and tissues, and participates in the regulation of nutrient absorption, tumor invasion and metastasis, and many other physiological and pathological processes (Ibegbu et al., 2009).

The role of CD26 in renal fibrosis (Takagaki et al., 2017), cardiac fibrosis (Bando and Murohara, 2016), hepatic fibrosis (Itou et al., 2013), wound healing and cutaneous diseases (Hu and Longaker, 2016; Patel et al., 2020) has been extensively discussed by recent reviews. Here, we would like to mainly address the advances of CD26 in skin fibrosis. The profibrotic nature of CD26 can be inspected from two intriguing observations: Dipeptidyl Peptidase-4 inhibitors (DPP4-Is) are novel oral hypoglycemics drugs used in clinical practice that work by blocking the enzymatic function of DPP-4 (Panchapakesan and Pollock, 2015). In a retrospective study, the occurrence rate of pathological scars (keloids and hypertrophic scars) after median sternotomy was significantly reduced in patients who have received DPP4-I treatment (Suwanai et al., 2020). Furthermore, CD26+ Fb population appeared more abundant in the human skin than in the gingiva, which may be associated with a better regeneration and less scarring property of gingiva (Mah et al., 2017). During the scarring process, fewer CD26+ Fbs was found in the regenerated gingival wounds as compared to the hypertrophic-like scars (Mah et al., 2017). Through rigorous lineage tracing experiments, Rinkevich et al. (2015) demonstrated that Engrailed-1 lineage-positive Fbs are the major cells responsible for matrix deposition in wound healing in mice skin. Moreover, cytometric screening identified CD26 as a surface marker for 94% of Engrailed-1 lineage-positive Fbs, and inhibition of CD26 with Diprotin A has resulted in mitigation of skin scarring (Rinkevich et al., 2015).

In contrast to the mice skin, studies of CD26 in human skin are relatively inconsistent (Philippeos et al., 2018; Tabib et al., 2018; Korosec et al., 2019; Vorstandlechner et al., 2020). Tabib et al. (2018) and Vorstandlechner et al. (2020) proposed that CD26+ Fbs that accumulate in both papillary and reticular layers have been demonstrated to be the major Fb subpopulation responsible for ECM assembly in normal skin and healing wounds in humans (Vorstandlechner et al., 2020; Worthen et al., 2020). In contrast, Philippeos et al. (2018) showed that CD26+ Fbs are enriched in the reticular dermis; while Korosec et al. (2019) believed that CD26+ Fbs are located in the papillary dermis but are also detectable in other dermal layers. Although consensus on the localization of CD26+ Fbs in human skin has yet to be reached, CD26+ Fbs derived from keloid, a typical fibrotic skin disorder after injury, demonstrated markedly elevated ability of cell proliferation and migration; greater expression of inflammatory and fibrotic factors such as TGF-β1, insulin growth factor-1 (IGF-1), and interleukin-6 (IL-6); and increased production of ECM components such as collagen 1, collagen 3 and fibronectin (Xin et al., 2017). In systemic sclerosis, CD26+ Fbs expressed high level of myofibroblast marker αSMA and collagen (Soare et al., 2020). The functional status of CD26 decides on the regulation of a series of cellular functions like cell proliferation, migration and collagen production. CD26 inhibition by genetic knockout or DPP4-I exerted potent antifibrotic effects in bleomycin-induced skin fibrosis (Soare et al., 2020).

It has been reported that the inhibition of CD26 exerted hypoglycaemic, anti-inflammatory, and antifibrotic effects (Panchapakesan and Pollock, 2015). The mechanism behind the antifibrotic role of CD26 is complex and has been investigated in several aspects. Primarily, it has been reported that CD26 blockage using established small molecular drugs would abrogate the classic TGF-β1 signaling pathways in hepatic (Kaji et al., 2014; Zhang et al., 2019; Khalil et al., 2020), pulmonary (Xu et al., 2018), renal (Seo et al., 2019; Kim et al., 2020), and adipose tissue (Marques et al., 2018) fibrosis. In high glucose culture environment, DPP4-I would decrease the expression of phosphorylated protein of the IGF/Akt/mTOR signaling pathway in hypertrophic scar-derived Fbs, inhibiting their *trans*-differentiation into myofibroblasts (Li et al., 2019). In addition, DPP4-I would reduce the level of inflammatory signals including NLR Family Pyrin Domain Containing 3 (NLRP3) inflammasome, endoplasmic reticulum (ER) stress and proinflammatory cytokine including IL-1β, IL-6, and tumor necrosis factor α (TNF-α) in the mice model of renal (Seo et al., 2019) and hepatic fibrosis (Jung et al., 2014). Apart from mediating the fibrotic and inflammatory pathways, DPP4-I also attenuated fibrogenesis by modulating the cellular status and phenotype including lipotoxicity-induced apoptosis (Lee et al., 2020) and endothelial–mesenchymal transition (EndMT) (Kanasaki et al., 2014; Suzuki et al., 2017; Xu et al., 2018). Aroor et al. (2015) reported the use of DPP4-I as a therapeutic rescue in patients with hepatic steatosis through the enhancement of mitochondrial glucose utilization and triacylglycerol secretion/export, thereby suppressing the accumulation of hepatic triacylglycerol and diacylglycerol. Taken together, these suggest that the metabolic regulation property of DPP4-I would be one of the main factors accounting for its antifibrotic effect.

**FIGURE 4:**
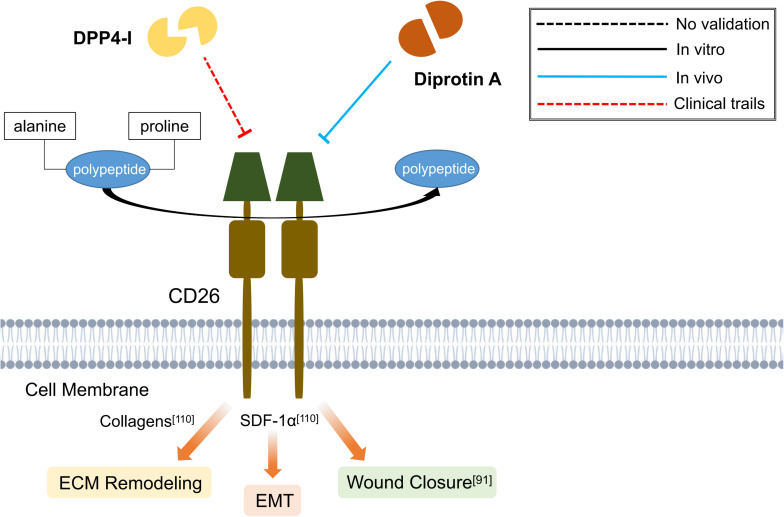
Schematic graph showing potential antifibrotic therapies targeting CD26. (DPP4-I, dipeptidyl peptidase-4 inhibitor; SDF-1α, stromal cell-derived factor 1α; EMT, epithelial-mesenchymal transition; ECM, extracellular matrix).

Until now, DPP4-I has been proven to exhibit prominent anti-fibrotic effects on liver (Jung et al., 2014; Kaji et al., 2014; Aroor et al., 2015; Zhang et al., 2019; Khalil et al., 2020; Lee et al., 2020), lung (Suzuki et al., 2017; Xu et al., 2018), kidney (Kanasaki et al., 2014; Gangadharan Komala et al., 2016; Uchii et al., 2016; Seo et al., 2019; Kim et al., 2020), adipose tissue (Marques et al., 2018) and skin (Long et al., 2018; Li et al., 2019; Soare et al., 2020). These studies signify the potential use of DPP4-I in combating fibrotic diseases (Figure 4). However, further clinical trials are still required to verify its effect. What’s more, the comparison of different types of DPP4-I regarding their antifibrotic effectiveness on various organ system is scarce in current researches. In addition, a more detailed knowledge concerning the dosage and adverse effects of a particular type of DPP4-I may be beneficial for future clinical application.

### CD36

CD36 (also known as scavenger receptor B2), is identified as a single chain transmembrane glycoprotein with two transmembrane domains and an extracellular region (Yang et al., 2017). The versatile function of CD36 is attributed to its wide range of ligands, which mainly includes the long-chain fatty acids, oxidized lipids and phospholipids, advanced oxidation protein products (AOPPs), thrombospondin and advanced glycation end products (AGEPs) (Yang et al., 2017). This ligand-receptor complex on the response element suggested the important role of CD36 in lipid metabolism, immunological response, inflammatory stress response, fibrosis and angiogenesis and arthrosclerosis (Febbraio et al., 2001; Sweetwyne and Murphy-Ullrich, 2012).

CD36 is expressed in multiple cells including renal tubule epithelial cells, podocytes, macrophages, microvascular endothelial cells and dermal Fbs (Febbraio et al., 2001; Philippeos et al., 2018). It has been reported that Lin-CD90 + CD36+ Fbs, which mainly located in the reticular dermis, possessed an inflammatory phenotype that included higher expression of αSMA, MGP, PPARγ; greater secretion of ECM and inflammatory factors; increased sensitivity upon interferon-γ (IFN-γ) stimulation; and stronger adipogenic differentiation (Philippeos et al., 2018; Korosec et al., 2019). In IPF, a group of CD36 + CD97+ Fbs has been identified in the remodeled areas of IPF tissue but with low expression of αSMA and ECM, suggesting that these Fbs are quiescent and are non-ECM producers in pulmonary fibrosis (Heinzelmann et al., 2018).

**FIGURE 5:**
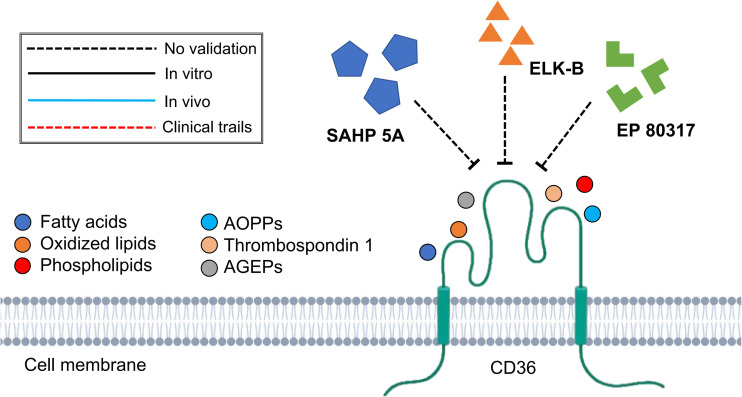
Schematic graph showing potential antifibrotic therapies targeting CD36. (AOPPs, advanced oxidative protein products; AGEPs, advanced glycation end products; SAHP 5A, synthetic amphipathic helical peptides 5A).

Although CD36 + Fbs have not shown fibrogenic phenotype, CD36 receptor would, however, associate with thrombospondin-1 (TSP-1)/latent TGFβ1 (L-TGFβ1) and facilitate the release of mature TGF-β1, initiating tissue fibrotic response (Yehualaeshet et al., 2000). The inhibition of this CD36/TSP-1/L-TGFβ1 regulatory pathway has been demonstrated to exert antifibrotic effects in lung (Yehualaeshet et al., 2000; Wang et al., 2009) and kidney (Yang et al., 2007; Pennathur et al., 2015) fibrotic diseases. On the other hand, the lipid transportation function of CD36 also contributed to tissue fibrogenesis. In tubular epithelial cell-specific CD36 overexpressed transgenic mice model, Kang et al. (2015) discovered the association of the increased long-chain fatty acid transportation with increasing αSMA and collagen 1 expression, which then facilitates the development of renal fibrosis. On the contrary, Rabinowitz and Mutlu (2019) and Zhao et al. (2019) found that the transplantation of CD36-overexpressed fibroblasts into the mouse skin would effectively reduce the radiation-induced skin fibrosis by activating fatty acid utilization while inhibiting glycolysis pathway. Therefore, there remains controversies concerning the relationship between fatty acid transportation function of CD36 and fibrosis as the choice of animal models and interventions would lead to completely distinct outcomes. On the other hand, the inflammation regulatory role of CD36 has also been reported in the CD36 expressing macrophages that have been reported to promote chronic kidney fibrogenesis by facilitating the activation of nuclear factor-κB (NF-κB) signaling and increasing oxidative stress (Okamura et al., 2009). The binding of lipoproteins, a biological ligand, to CD36 allows the activation of Toll-like receptors (TLRs), Sodium–Potassium Adenosine Triphosphatase (Na+/K+ ATPase), the NLRP3 inflammasome, protein kinase C-nicotinamide adenine dinucleotide phosphate oxidase (NAPDH) oxidase, Src-family kinases (Scr/Lyn/Fyn), mitogen-activated protein kinases, and TGF-β signaling pathways (Yang et al., 2017). As such, synthetic amphipathic helical peptides (SAHPs) can mimic the domain of lipoprotein and bind to the CD36 without exerting activation effects (Souza et al., 2016). In mice models of nephrectomy and angiotensin II-induced chronic renal fibrosis, SAHP 5A decreased the expression of inflammation-associated genes and attenuated the progression of glomerular sclerosis and interstitial fibrosis, thereby providing renal protection. However, due to the relatively low selectivity of SAHP 5A which also targets other scavenger receptors (SR BI/II), a more specific CD36 binding SAHP, namely ELK-SAHPs, has been synthesized (Yang et al., 2017). Among them, ELK-B has been shown to improve lung function in the mice model of sepsis by effectively reducing pulmonary infiltration (Bocharov et al., 2016). Another strategy to inhibit the function of CD36 is by using hexapeptide growth hormone-releasing peptides (GHRPs) analog, namely EP 80317. EP 80317 has been found to protect against atherosclerosis progression (Marleau et al., 2005) and myocardial ischemia/reperfusion injury (Bessi et al., 2012). Nevertheless, the above therapies targeting the CD36 are currently under preclinical animal studies and have only been validated in inflammatory and fibrotic diseases of the kidney, lung and heart. Further studies are warranted to verify the anti-fibrosis effect of targeting CD36 in fibrotic diseases of other organ systems (Figure 5). Considering the pleiotropic effects of CD36, a deeper knowledge regarding the cellular specificity of CD36 function would be necessary for a better development of pharmaceutical strategy (Yang et al., 2017).

### CD39

CD39 (also known as ectonucleoside triphosphate diphosphohydrolase-1, ENTPD1) is an integral cell membrane glycoprotein that exhibit extracellular nucleotide hydrolase activity (Faas et al., 2017). CD39 converts extracellular adenosine triphosphate (ATP) to the adenosine monophosphate (AMP), which is then converted to adenosine (ADO) by CD73 (Faas et al., 2017). The phosphohydrolysis of ATP to AMP is a rate-limiting step in ADO generation (Faas et al., 2017). Philippeos et al. (2018) first uncovered the use of CD39 as a marker for papillary Fb subtype and demonstrated that CD39+ Fbs exhibited higher proliferation rate and can better support epidermal growth in comparison with CD36+ reticular Fbs. On the contrary, papillary CD39+ Fbs showed lower expression level of ECM and inflammatory cytokine, lower adipogenic capacity and are less responsive to inflammatory signals such as IFN-γ, as compared to CD36+ reticular Fbs (Philippeos et al., 2018).

At present, such studies investigating the cellular function of CD39+ Fbs are scarce, especially regarding their role in the process of tissue fibrosis. However, the role of CD39 protein *per se* in fibrotic diseases have been studied and showed competing results. On one hand, CD39 hyperfunction caused the accumulation of extracellular ADO and facilitated the activation of ADO signaling pathway, resulting in tissue fibrosis (Roberts et al., 2017). In renal ischemia-reperfusion injury models, overexpression of CD39 in the transgenic mice showed significantly more severe renal fibrosis via upregulating ADO and stimulating profibrotic downstream pathways through interaction with adenosine A2B receptors (Roberts et al., 2017). Consistently, antifibrogenic effects of CD39 depletion have been observed in bleomycin-induced skin fibrosis (Fernández et al., 2013) and cyclosporin-induced pancreatitis (Künzli et al., 2008) models, probably through modulating the accumulation of ADO (Fernández et al., 2013) and the expression of profibrotic factors (Fernández et al., 2013)and pro-collagen proteins (Künzli et al., 2008). However, other studies argued that the fibrogenic nature of CD39 appeared to be quite inconsistent between organ systems and fibrosis induction methods (Wang et al., 2012; Roberts et al., 2016; Rothweiler et al., 2019). For instance, in the Adriamycin-induced nephropathy model, transplantation of CD39 overexpressing CD25+ regulatory T cells showed renal protective effects by attenuating ATP-induced cell apoptosis and inflammation (Wang et al., 2012). Moreover, global CD39 overexpression showed no difference in fibrotic parameters in the unilateral ureteric obstructive mice model, which is inconsistent with that reported in the renal ischemia-reperfusion injury mice models (Roberts et al., 2016; Roberts et al., 2017). Similar discordance has also been observed in hepatic and biliary system where global CD39 depletion attenuated pancreatitis related fibrosis (Künzli et al., 2008) yet exacerbated 3,5-diethoxycarbonyl-1,4-dihydrocollidine (DDC) induced biliary fibrosis (Rothweiler et al., 2019).

The reason for the inconsistency between these studies may be various: (1) Firstly, CD39-mediated purinergic signaling pathway is relatively intricate. Both P1 receptor sensing ADO and P2 receptor sensing ATP have been reported for their pro-fibrotic role (Burnstock et al., 2012; Ferrari et al., 2016). The hyperfunction of CD39 would result in decreased ATP signaling and enhanced ADO signaling, so the outcome in different tissues or cells is reliant on the superposition of the activation states of the two kinds of receptors (Roberts et al., 2016); (2) Secondly, due to the diverse distribution patterns of P1 and P2 receptors and their subtypes in different tissues, the functional changes of CD39 would serve different outcomes in different tissues (Ferrari et al., 2016); (3) Finally, the underlying pathogenesis of fibrotic diseases in different organ systems are distinct, and CD39 may not necessarily be crucial for every type of tissue fibrosis.

**FIGURE 6:**
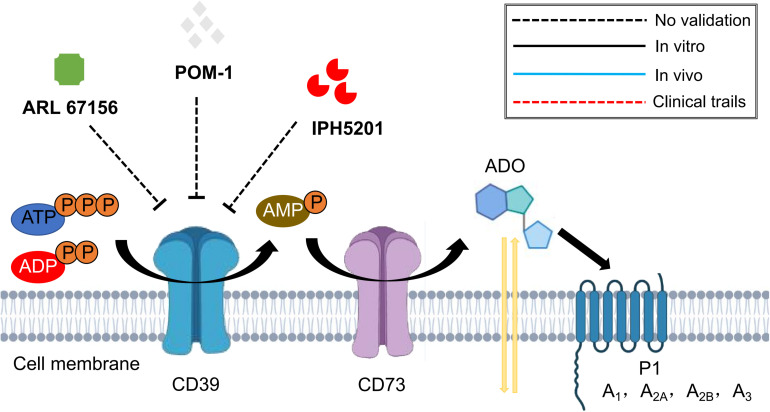
Schematic graph showing potential antifibrotic therapies targeting CD39. (ATP, adenosine triphosphate; ADP, adenosine diphosphate; AMP, adenosine monophosphate; ADO, adenosine).

To date, several components are available for CD39 targeting, which include the small molecular drugs ARL 67156 trisodium salt and POM-1 (Fang et al., 2016; Yang et al., 2020), and the monoclonal antibody IPH5201 (Perrot et al., 2019). These targeted treatments have shown promising safety profile and therapeutic potential in the regulation of inflammatory response and tumor microenvironment (Fang et al., 2016; Perrot et al., 2019; Yang et al., 2020). However, there is still a lack of studies evaluating their application in fibrotic process, and is definitely worthy of further exploration (Figure 6).

## Summary and Perspectives

The advances of high-throughput sequencing technologies have greatly facilitated the investigation on tissue fibrosis, a multiplexed process involving different cell types and factors. In this review, we focused on several surface markers of human dermal Fbs identified in recent years and discussed their respective roles as lineage markers or functional entity in fibrotic diseases. Since the initial discovery, more references are available concerning the role of Fbs defined by CD90, FAP, PDGFRα/PDGFRβ and CD26 solely, or in combination with other markers. However, only Fbs defined by PDGFRα/PDGFRβ or CD26 showed consistent fibrogenic potential between studies and appeared in accordance with the molecular function of surface markers *per se* (see Tables 1, 2). As there are limited literatures on the more recently identified Fb markers like CD36 and CD39, this demands further researches to investigate their respective roles in fibrotic diseases. Moreover, the function of both of these membrane bound proteins showed comprehensive regulatory network in tissue fibrosis and distinct results across different organs, complicating the interpretation of their biological functions. Surface membrane proteins are considered as the ideal therapeutic targets as they own extracellular domains for ligand binding (Bansal et al., 2016). However, the surface marker does not necessarily account for all of the functional dysregulation of certain Fb subtype. Therefore, an in depth profiling to investigate the key functional targets that characterize Fb subtypes is still required to provide a better and more effective targeting strategy.

**TABLE 1:** The function of Fb subtypes in tissue fibrosis.

**Fb subtypes**	**Models and diseases**	**description**	**Effect**
CD90 + CD34−	Human scars (Ho et al., 2019)	Colocalize with αSMA and procollagen-1	Fibrogenic
CD90 + podoplanin + CD34−	Human SSC (Nazari et al., 2016)	Expanded cell population	Fibrogenic
CD90 + CD45−	Murine cholestatic liver injury (Katsumata et al., 2017)	High level of expression of αSMA, collagen-1 and TIMP-1	Fibrogenic
PDGFRa + CD9^high^	Murine obesity-induced WAT fibrosis (Marcelin et al., 2017)	Give rise to profibrotic cells; modulate omental WAT fibrogenesis	Fibrogenic
PDGFRα + TPPP3+	Murine tendon injury (Harvey et al., 2019)	Fibrotic scars formation in healing tendons	Fibrogenic
PDGFRα+	Human cirrhotic liver; murine CCl4-induced liver fibrosis (Ramachandran et al., 2019)	Expanded cell population in fibrotic niche	Fibrogenic
PDGFRα+	Murine ischemic injury (Santini et al., 2020)	Promotion of skeletal muscle fibrosis upon ischemic injury.	Fibrogenic
PDGFRα + Sca1 + CD45−	Murine DMD (Ieronimakis et al., 2016)	Expanded cell population; major matrix-forming Fbs	Fibrogenic
PDGFRα + ADAM12+	Murine muscle and skin injury (Dulauroy et al., 2012)	Major fraction of collagen-overproducing cells	Fibrogenic
FAP + CD90−	Human skin (Korosec et al., 2019)	High proliferative potential; low adipogenic potential; enriched in the papillary dermis	Non-fibrogenic
FAP+	Human and rat myocardial infarction (Tillmanns et al., 2015)	Located in peri-infarct area with co-expression of prolyl-4-hydroxylase β, αSMA, and vimentin	Fibrogenic
CD26 + Sca1−	Murine skin (Driskell et al., 2013; Philippeos et al., 2018)	Papillary Fbs with upregulation of Wnt pathway related genes (Philippeos et al., 2018); large fraction of dermal Fbs in adult mice (Driskell et al., 2013)	Non-fibrogenic
CD26+	Murine (Rinkevich et al., 2015) and human wound (Vorstandlechner et al., 2020; Worthen et al., 2020)	Major scar-forming Engrailed1+ Fbs (Rinkevich et al., 2015); main ECM-producing Fbs during the remodeling phase of wound healing (Vorstandlechner et al., 2020; Worthen et al., 2020)	Fibrogenic
CD26+	Human keloid (Xin et al., 2017)	Expended cell population; upregulated proliferation, invasion and expression of profibrotic genes	Fibrogenic
Lin-CD90 + CD36+	Human skin (Philippeos et al., 2018; Korosec et al., 2019)	Localize in lower reticular dermis and hypodermis; high adipogenic potential; high expression of ECM and inflammatory related genes	Inflammatory
CD36 + CD97+	Human IPF (Heinzelmann et al., 2018)	Low cell proliferation rate; low expression of αSMA and ECM	Non-fibrogenic
Lin-CD90 + CD39+	Human and mice skin (Philippeos et al., 2018; Korosec et al., 2019)	Enriched in papillary dermis; low adipogenic potential; low expression of ECM and inflammatory related genes	Non-fibrogenic

*SSC, systemic sclerosis; WAT, white adipose tissue; DMD, duchenne muscular dystrophy; IPF, idiopathic pulmonary fibrosis.*

**TABLE 2:** The function of surface markers *per se* in tissue fibrosis.

**Markers**	**Diseases**	**Intervention and models**	**Mechanism**	**Effect**
CD90	Periprosthetic capsular (Hansen et al., 2017)	Lentiviral depletion; scar-derived Fbs	Collagen production; myofibroblast activation	Profibrotic
	IPF (Fiore et al., 2015)	Plasmid overexpression and lentiviral knockdown; human and mice lung Fbs	CD90 and αvβ3 integrins interaction; mechanosensitive Rho signaling pathway	Profibrotic
	Lung development (Nicola et al., 2009)	Thy-1^–/–^ transgenic mice	Cell proliferation; production of collagen and elastin; TGF-β signaling pathway	Antifibrotic
	IPF (Sanders et al., 2008)	DNA methyltransferase Inhibitors; rat and human lung Fbs	Methylation-regulated expression of CD90; myofibroblast activation	Antifibrotic
PDGFRα/β	Systemic fibrosis (Olson and Soriano, 2009)	PDGFRα knockin mice in Ink4a/Arf-deficient background	Growth of connective tissue; collagen production	Profibrotic
	Liver fibrosis (Hayes et al., 2014)	PDGFRα GFP reporter mice; PDGFRα heterozygous mice	Expression of PDGFRα and fibrogenic genes; collagen deposition	Profibrotic
	Liver fibrosis (Kocabayoglu et al., 2015)	PDGFRβ*^*fl/fl*^*: GFAP^*Cre*^; PDGFRβ*^*betaJ/*+^* : GFAP^*Cre*^	Expression of collagen and αSMA; ERK, AKT, and NF-kB signaling pathways	Profibrotic
	Liver fibrosis (Czochra et al., 2006)	Transgenic mice overexpressing PDGF-B in the liver	Myofibroblast activation; collagen deposition; production of MMP-2, MMP-9, and TIMP-1	Profibrotic
FAP	Lung fibrosis (Kimura et al., 2019)	FAP targeting CAR-T cells; FAP knockout mice	Collagen production; myofibroblast activation; leukocyte infiltration	Contradictory
	Crohn’s disease (Truffi et al., 2018)	FAP targeting antibody; primary mucosal myofibroblasts	Collagen and TIMP-1 production; myofibroblast migration	Profibrotic
	Liver injury (Wang et al., 2005)	Plasmid overexpression; HSC cell line	Cell adhesion, migration, invasion and apoptosis	Profibrotic
CD26	Systemic sclerosis (Soare et al., 2020)	DPP4-knockout and DPP4-I; murine model of bleomycin-induced fibrosis	Cell proliferation and migration; expression of collagen and contractile proteins; TGF-β/ERK signaling pathway	Profibrotic
	Hypertrophic scar (Li et al., 2019)	DPP4-I; HSF	Myofibroblast differentiation; IGF/Akt/mTOR signaling pathway	Profibrotic
	Keloid (Thielitz et al., 2008)	Lys[Z(NO_2_)]-thiazolidide and Lys[Z(NO_2_)]-pyrrolidide; keloid-derived Fbs	Cell proliferation; expression of TGF-β and procollagen type I; mitogen-activated protein kinases pp38 and pERK1/2 signaling pathway	Profibrotic
	Diabetic Wound Healing (Long et al., 2018)	DPP4-I; human fibroblast cell line; murine model of diabetic wounds; patients with refractory ulcers	Collagen deposition; SDF-1α production; keratinocyte EMT	Profibrotic
CD36	Pulmonary fibrosis (Yehualaeshet et al., 2000)	CD36 inhibitory peptide; rt model of bleomycin-induced fibrosis	Production of TGF-β1, inflammatory factors, and ECM	profibrotic
	Skin fibrosis (Rabinowitz and Mutlu, 2019; Zhao et al., 2019)	Transplantation of CD36^*high*^ Fb or CD36^*KO*^ Fb; murine model of radiation-induced skin fibrosis	Fatty acid oxidation; degradation of collagen-1; ECM accumulation	Antifibrotic
	Renal fibrosis (Kang et al., 2015)	Pax8rtTA/TRE-CD36 double-transgenic mice	Intracellular lipid accumulation; expression of collagen-1 and αSMA	Profibrotic
	Renal fibrosis (Souza et al., 2016)	CD36 antagonist (apolipoprotein AI-mimetic peptide 5A); murine model of unilateral ureteral obstruction	Macrophage infiltration; expression of inflammasome genes; interstitial fibrosis	Profibrotic
	Renal fibrosis (Okamura et al., 2009)	CD36^–/–^ transgenic mice	Regulation of oxidative stress; myofibroblast activation; NF-κB signaling pathway	Profibrotic
	Chronic kidney injury (Pennathur et al., 2015)	CD36-deficient mice	Production of intracellular bioactive oxidized lipids, TNF-α and TGF-β1	Profibrotic
	Renal tubule fibrosis (Yang et al., 2007)	siRNA knockdown; LLCPK1 cell line	Albumin production; expression of TGF-β1 and fibronectin	Profibrotic
	Lung fibrosis (Wang et al., 2009)	Lentiviral depletion; rat silicosis model	Activation of L-TGF-β1; production of hydroxyproline and ECM	Profibrotic
CD39	Chronic renal Fibrosis (Roberts et al., 2017)	CD39 over-expressing transgenic mice	Adenosine generation	Profibrotic
	Skin fibrosis (Fernández et al., 2013)	CD39 knockout mice	Adenosine generation; production of collagen and profibrotic cytokines; myofibroblast activation	Profibrotic
	Pancreatitis (Künzli et al., 2008)	CD39-null mice; PSC with CD39 depletion	Cell proliferation; expression of procollagen-α1 and IFN-γ	Profibrotic
	Chronic renal injury (Wang et al., 2012)	CD39 over-expressing transgenic mice	CD25+ Treg cells; production of urinary protein and serum creatinine level	Antifibrotic
	Chronic kidney injury (Roberts et al., 2016)	CD39 over-expressing transgenic mice	No protective effects on renal fibrosis	Non-effective
	Sclerosing cholangitis (Rothweiler et al., 2019)	Global or myeloid-specific CD39-deficient mice	Collagen production; expression of profibrotic genes *Tgf-β1*, *Tnf-α*, and *α-Sma*	Antifibrotic

*IPF, idiopathic pulmonary fibrosis; HSC, hepatic stellate cells; PSC, pancreatic stellate cells; HSF, hypertrophic scar-derived fibroblasts; EMT, epithelial-mesenchymal transition.*

At the same time, there are also some methodological and technical difficulties at this stage: (1) Most Fb subtype surface markers are co-expressed, thus it is challenging to manipulate and to perform research on a specific group of Fbs (Ascensión et al., 2020). (2) It has been suggested that the phenotype of Fb subtypes requires a specific *in vivo* microenvironment (Korosec et al., 2019),which is, however, hard to maintain after *in vitro* culture, indicating that functional profiling directly after cell isolation might provide more reliable information than *in vitro* experiments. (3) Many newly discovered Fb subtypes still lack verification on their selective tissue localization. Emerging techniques like imaging mass cytometry and spatial transcriptomics (Giesen et al., 2014; Vickovic et al., 2019) can provide the possibility for combined analysis of functional sequencing and spatial profiling data. (4) Since most of mechanistic studies of Fb subtypes so far are based on animal models, in view of the limited conservation of Fb subtypes between human and mice skin (Philippeos et al., 2018), verification of the function of Fb subtypes or related therapies using human samples, cells or humanized animals is undoubtedly essential and should be the main focus in upcoming studies. (5) The investigation on Fb subtypes in human subjects would definitely be a challenge due to the multifactorial causation of the disease development and tremendous heterogeneity that exists among patients. Thus, it is suggested to pay particular attention on strict selection of homogeneous clinical samples. Besides, analyzing samples from different points in the disease course to reduce bias caused by focusing only on the healthy and diseased states, might provide additional hints on disease progression (Shaw and Rognoni, 2020). (6) The current understanding of the origin of different Fb subtypes remains incomplete. Recent studies demonstrated the conversion of circulating myeloid cells to Fbs during wound healing, indicating a dedicate regulation of Fb fate switching (Sinha et al., 2018; Guerrero-Juarez et al., 2019). Further investigation into the evolution of Fb subtypes and factors influencing the Fbs plasticity could facilitate the development of potential therapeutic approaches in treating fibrotic diseases.

In the future, a more comprehensive transcriptomic, proteomic or genome-wide epigenetic profiling of Fb subtypes defined by different surface markers would be instrumental for a more in-depth understanding of the evolution and functional characteristics of each Fb subtype in tissue fibrosis. Hopefully, a more precise dissection of phenotype features and functional regulation of Fb subtypes would bring greater clinical insights for targeted treatment strategies for fibrotic diseases.

## Author Contributions

XH, QL, and TZ: conception and design. XH, YK, SG, CH, DS, and HM: collection and assembly of data. XH and YK: data analysis and interpretation and manuscript revision. XH, CH, DS, HM, and SG: graphic illustration. All authors wrote and approved the final manuscript.

## Conflict of Interest

The authors declare that the research was conducted in the absence of any commercial or financial relationships that could be construed as a potential conflict of interest.

## References

[B1] Acharya P. S. Zukas A. Chandan V. Katzenstein A. L. Puré E. (2006). Fibroblast activation protein: a serine protease expressed at the remodeling interface in idiopathic pulmonary fibrosis. *Hum. Pathol.* 37 352–360. 10.1016/j.humpath.2005.11.020 16613331

[B2] Aghajanian H. Kimura T. Rurik J. G. Hancock A. S. Leibowitz M. S. Li L. (2019). Targeting cardiac fibrosis with engineered T cells. *Nature* 573 430–433. 31511695 10.1038/s41586-019-1546-z PMC6752964

[B3] Akhmetshina A. Dees C. Pileckyte M. Maurer B. Axmann R. Jüngel A. (2008). Dual inhibition of c-abl and PDGF receptor signaling by dasatinib and nilotinib for the treatment of dermal fibrosis. *FASEB J.* 22 2214–2222. 10.1096/fj.07-105627 18326784

[B4] Akhmetshina A. Venalis P. Dees C. Busch N. Zwerina J. Schett G. (2009). Treatment with imatinib prevents fibrosis in different preclinical models of systemic sclerosis and induces regression of established fibrosis. *Arthrit. Rheumat.* 60 219–224. 10.1002/art.24186 19116940

[B5] Aroor A. R. Habibi J. Ford D. A. Nistala R. Lastra G. Manrique C. (2015). Dipeptidyl peptidase-4 inhibition ameliorates Western diet-induced hepatic steatosis and insulin resistance through hepatic lipid remodeling and modulation of hepatic mitochondrial function. *Diabetes* 64 1988–2001. 10.2337/db14-0804 25605806 PMC4439570

[B6] Ascensión A. M. Fuertes-Álvarez S. Ibañez-Solé O. Izeta A. Araúzo-Bravo M. J. (2020). Human dermal fibroblast subpopulations are conserved across single-Cell RNA sequencing studies. *J. Invest. Dermatol.* [Epub ahead of print]. 10.1016/j.jid.2020.11.028 33385399

[B7] Avery D. Govindaraju P. Jacob M. Todd L. Monslow J. Puré E. (2018). Extracellular matrix directs phenotypic heterogeneity of activated fibroblasts. *Matrix Biol.* 67 90–106. 10.1016/j.matbio.2017.12.003 29248556 PMC5910258

[B8] Bando Y. K. Murohara T. (2016). Heart failure as a comorbidity of diabetes: role of dipeptidyl peptidase 4. *J. Atheroscl. Thromb.* 23 147–154. 10.5551/jat.33225 26607352

[B9] Bansal R. Nagórniewicz B. Prakash J. (2016). Clinical advancements in the targeted therapies against liver fibrosis. *Mediat. Inflamm.* 2016:7629724. 10.1155/2016/7629724 PMC5143744 27999454

[B10] Bessi V. L. Labbé S. M. Huynh D. N. Ménard L. Jossart C. Febbraio M. (2012). EP 80317, a selective CD36 ligand, shows cardioprotective effects against post-ischaemic myocardial damage in mice. *Cardiovasc. Res.* 96 99–108. 10.1093/cvr/cvs225 22787133

[B11] Bocharov A. V. Wu T. Baranova I. N. Birukova A. A. Sviridov D. Vishnyakova T. G. (2016). Synthetic amphipathic helical peptides targeting CD36 attenuate lipopolysaccharide-induced inflammation and acute lung injury. *J. Immunol.* 197 611–619. 27316682 10.4049/jimmunol.1401028

[B12] Borkham-Kamphorst E. Stoll D. Gressner A. M. Weiskirchen R. (2004). Inhibitory effect of soluble PDGF-beta receptor in culture-activated hepatic stellate cells. *Biochem. Biophys. Res. Commun.* 317 451–462. 10.1016/j.bbrc.2004.03.064 15063779

[B13] Burnstock G. Knight G. E. Greig A. V. (2012). Purinergic signaling in healthy and diseased skin. *J. Invest. Dermatol.* 132(3 Pt 1), 526–546. 10.1038/jid.2011.344 22158558

[B14] Busek P. Mateu R. Zubal M. Kotackova L. Sedo A. (2018). Targeting fibroblast activation protein in cancer - prospects and caveats. *Front. Biosci.* 23:1933–1968. 10.2741/4682 29772538

[B15] Chen W. C. Chang Y. S. Hsu H. P. Yen M. C. Huang H. L. Cho C. Y. (2015). Therapeutics targeting CD90-integrin-AMPK-CD133 signal axis in liver cancer. *Oncotarget* 6 42923–42937. 10.18632/oncotarget.5976 26556861 PMC4767481

[B16] Croft A. P. Campos J. Jansen K. Turner J. D. Marshall J. Attar M. (2019). Distinct fibroblast subsets drive inflammation and damage in arthritis. *Nature* 570 246–251. 10.1038/s41586-019-1263-7 31142839 PMC6690841

[B17] Czochra P. Klopcic B. Meyer E. Herkel J. Garcia-Lazaro J. F. Thieringer F. (2006). Liver fibrosis induced by hepatic overexpression of PDGF-B in transgenic mice. *J. Hepatol.* 45 419–428. 10.1016/j.jhep.2006.04.010 16842882

[B18] Dienus K. Bayat A. Gilmore B. F. Seifert O. (2010). Increased expression of fibroblast activation protein-alpha in keloid fibroblasts: implications for development of a novel treatment option. *Arch. Dermatol. Res.* 302 725–731. 10.1007/s00403-010-1084-x 20872224

[B19] Distler J. H. Jüngel A. Huber L. C. Schulze-Horsel U. Zwerina J. Gay R. E. (2007). Imatinib mesylate reduces production of extracellular matrix and prevents development of experimental dermal fibrosis. *Arthrit. Rheumat.* 56 311–322. 10.1002/art.22314 17195235

[B20] Driskell R. R. Lichtenberger B. M. Hoste E. Kretzschmar K. Simons B. D. Charalambous M. (2013). Distinct fibroblast lineages determine dermal architecture in skin development and repair. *Nature* 504 277–281. 10.1038/nature12783 24336287 PMC3868929

[B21] Driskell R. R. Watt F. M. (2015). Understanding fibroblast heterogeneity in the skin. *Trends Cell Biol.* 25 92–99. 10.1016/j.tcb.2014.10.001 25455110

[B22] Dulauroy S. Di Carlo S. E. Langa F. Eberl G. Peduto L. (2012). Lineage tracing and genetic ablation of ADAM12(+) perivascular cells identify a major source of profibrotic cells during acute tissue injury. *Nat. Med.* 18 1262–1270. 10.1038/nm.2848 22842476

[B23] Egger C. Cannet C. Gérard C. Suply T. Ksiazek I. Jarman E. (2017). Effects of the fibroblast activation protein inhibitor, PT100, in a murine model of pulmonary fibrosis. *Eur. J. Pharmacol.* 809 64–72. 10.1016/j.ejphar.2017.05.022 28506908

[B24] Faas M. M. Sáez T. de Vos P. Extracellular A. T. P. (2017). and adenosine: the Yin and Yang in immune responses? *Mol. Aspects Med.* 55 9–19. 10.1016/j.mam.2017.01.002 28093236

[B25] Fang F. Yu M. Cavanagh M. M. Hutter Saunders J. Qi Q. Ye Z. (2016). Expression of CD39 on activated T cells impairs their survival in older individuals. *Cell Rep.* 14 1218–1231. 10.1016/j.celrep.2016.01.002 26832412 PMC4851554

[B26] Febbraio M. Hajjar D. P. Silverstein R. L. (2001). CD36: a class B scavenger receptor involved in angiogenesis, atherosclerosis, inflammation, and lipid metabolism. *J. Clin. Invest.* 108 785–791. 10.1172/jci14006 11560944 PMC200943

[B27] Fernández P. Perez-Aso M. Smith G. Wilder T. Trzaska S. Chiriboga L. (2013). Extracellular generation of adenosine by the ectonucleotidases CD39 and CD73 promotes dermal fibrosis. *Am. J. Pathol.* 183 1740–1746. 10.1016/j.ajpath.2013.08.024 24266925 PMC5362691

[B28] Ferrari D. Gambari R. Idzko M. Müller T. Albanesi C. Pastore S. (2016). Purinergic signaling in scarring. *FASEB J.* 30 3–12. 10.1096/fj.15-274563 26333425 PMC4684510

[B29] Fiore V. F. Strane P. W. Bryksin A. V. White E. S. Hagood J. S. Barker T. H. (2015). Conformational coupling of integrin and Thy-1 regulates Fyn priming and fibroblast mechanotransduction. *J. Cell Biol.* 211 173–190. 10.1083/jcb.201505007 26459603 PMC4602038

[B30] Fleetwood K. McCool R. Glanville J. Edwards S. C. Gsteiger S. Daigl M. (2017). Systematic review and network meta-analysis of idiopathic pulmonary fibrosis treatments. *J. Manag. Care Special. Pharm.* 23(3–b Suppl.), S5–S16. 10.18553/jmcp.2017.23.3-b.s5 PMC10410677 28287346

[B31] Fraticelli P. Gabrielli B. Pomponio G. Valentini G. Bosello S. Riboldi P. (2014). Low-dose oral imatinib in the treatment of systemic sclerosis interstitial lung disease unresponsive to cyclophosphamide: a phase II pilot study. *Arthrit. Res. Ther.* 16:R144. 10.1186/ar4606 PMC4227120 25007944

[B32] Gangadharan Komala M. Gross S. Zaky A. Pollock C. Panchapakesan U. (2016). Saxagliptin reduces renal tubulointerstitial inflammation, hypertrophy and fibrosis in diabetes. *Nephrology* 21 423–431. 10.1111/nep.12618 26375854

[B33] Gerber E. E. Gallo E. M. Fontana S. C. Davis E. C. Wigley F. M. Huso D. L. (2013). Integrin-modulating therapy prevents fibrosis and autoimmunity in mouse models of scleroderma. *Nature* 503 126–130. 10.1038/nature12614 24107997 PMC3992987

[B34] Giesen C. Wang H. A. Schapiro D. Zivanovic N. Jacobs A. Hattendorf B. (2014). Highly multiplexed imaging of tumor tissues with subcellular resolution by mass cytometry. *Nat. Methods* 11 417–422. 10.1038/nmeth.2869 24584193

[B35] Gilmore B. F. Lynas J. F. Scott C. J. McGoohan C. Martin L. Walker B. (2006). Dipeptide proline diphenyl phosphonates are potent, irreversible inhibitors of seprase (FAPalpha). *Biochem. Biophys. Res. Commun.* 346 436–446. 10.1016/j.bbrc.2006.05.175 16769036

[B36] Gordon J. Udeh U. Doobay K. Magro C. Wildman H. Davids M. (2014). Imatinib mesylate (Gleevec^TM^) in the treatment of diffuse cutaneous systemic sclerosis: results of a 24-month open label, extension phase, single-centre trial. *Clin. Exp. Rheumatol.* 32(6 Suppl. 86), 189–193. 25152211

[B37] Guerrero-Juarez C. F. Dedhia P. H. Jin S. Ruiz-Vega R. Ma D. Liu Y. (2019). Single-cell analysis reveals fibroblast heterogeneity and myeloid-derived adipocyte progenitors in murine skin wounds. *Nat. Commun.* 10:650. 10.1038/s41467-018-08247-x PMC6368572 30737373

[B38] Hansen T. C. Woeller C. F. Lacy S. H. Koltz P. F. Langstein H. N. Phipps R. P. (2017). Thy1 (CD90) expression is elevated in radiation-induced periprosthetic capsular contracture: implication for novel therapeutics. *Plast. Reconstruct. Surg.* 140 316–326. 10.1097/prs.0000000000003542 28746279 PMC5531053

[B39] Hao Z. M. Fan X. B. Li S. Lv Y. F. Su H. Q. Jiang H. P. (2012). Vaccination with platelet-derived growth factor B kinoids inhibits CCl4-induced hepatic fibrosis in mice. *J. Pharmacol. Exp. Therap.* 342 835–842. 10.1124/jpet.112.194357 22711911

[B40] Harvey T. Flamenco S. Fan C. M. (2019). A Tppp3(+)Pdgfra(+) tendon stem cell population contributes to regeneration and reveals a shared role for PDGF signalling in regeneration and fibrosis. *Nat. Cell Biol.* 21 1490–1503. 10.1038/s41556-019-0417-z 31768046 PMC6895435

[B41] Hayes B. J. Riehle K. J. Shimizu-Albergine M. Bauer R. L. Hudkins K. L. Johansson F. (2014). Activation of platelet-derived growth factor receptor alpha contributes to liver fibrosis. *PLoS One* 9:e92925. 10.1371/journal.pone.0092925 24667490 PMC3965491

[B42] Heinzelmann K. Lehmann M. Gerckens M. Noskovičová N. Frankenberger M. Lindner M. (2018). Cell-surface phenotyping identifies CD36 and CD97 as novel markers of fibroblast quiescence in lung fibrosis. *Am. J. Physiol. Lung Cell. Mol. Physiol.* 315 L682–L696. 29952218 10.1152/ajplung.00439.2017

[B43] Higashi T. Friedman S. L. Hoshida Y. (2017). Hepatic stellate cells as key target in liver fibrosis. *Adv. Drug Deliv. Rev.* 121 27–42. 10.1016/j.addr.2017.05.007 28506744 PMC5682243

[B44] Ho J. D. Chung H. J. Ms Barron A. Ho D. A. Sahni D. Browning J. L. (2019). Extensive CD34-to-CD90 fibroblast transition defines regions of cutaneous reparative, hypertrophic, and keloidal scarring. *Am. J. Dermatopathol.* 41 16–28. 10.1097/dad.0000000000001254 30320623

[B45] Hu M. S. Longaker M. T. (2016). Dipeptidyl peptidase-4, wound healing, scarring, and fibrosis. *Plast. Reconstruct. Surg.* 138 1026–1031. 10.1097/prs.0000000000002634 27782998

[B46] Ibegbu C. C. Xu Y. X. Fillos D. Radziewicz H. Grakoui A. Kourtis A. P. (2009). Differential expression of CD26 on virus-specific CD8(+) T cells during active, latent and resolved infection. *Immunology* 126 346–353. 10.1111/j.1365-2567.2008.02899.x 18657205 PMC2669815

[B47] Ieronimakis N. Hays A. Prasad A. Janebodin K. Duffield J. S. Reyes M. (2016). PDGFRα signalling promotes fibrogenic responses in collagen-producing cells in Duchenne muscular dystrophy. *J. Pathol.* 240 410–424. 10.1002/path.4801 27569721 PMC5113675

[B48] Ishiura Y. Kotani N. Yamashita R. Yamamoto H. Kozutsumi Y. Honke K. (2010). Anomalous expression of Thy1 (CD90) in B-cell lymphoma cells and proliferation inhibition by anti-Thy1 antibody treatment. *Biochem. Biophys. Res. Commun.* 396 329–334. 10.1016/j.bbrc.2010.04.092 20403334

[B49] Itou M. Kawaguchi T. Taniguchi E. Sata M. (2013). Dipeptidyl peptidase-4: a key player in chronic liver disease. *World J. Gastroenterol.* 19 2298–2306. 23613622 10.3748/wjg.v19.i15.2298 PMC3631980

[B50] Jacob M. Chang L. Puré E. (2012). Fibroblast activation protein in remodeling tissues. *Curr. Mol. Med.* 12 1220–1243. 10.2174/156652412803833607 22834826

[B51] Jiang D. Rinkevich Y. (2018). Defining skin fibroblastic cell types beyond CD90. *Front. Cell Dev. Biol.* 6:133. 10.3389/fcell.2018.00133 30406099 PMC6204438

[B52] Jiang D. Rinkevich Y. (2020). Scars or regeneration?-Dermal fibroblasts as drivers of diverse skin wound responses. *Int. J. Mol. Sci.* 21:617. 10.3390/ijms21020617 31963533 PMC7014275

[B53] Jung Y. A. Choi Y. K. Jung G. S. Seo H. Y. Kim H. S. Jang B. K. (2014). Sitagliptin attenuates methionine/choline-deficient diet-induced steatohepatitis. *Diabetes Res. Clin. Pract.* 105 47–57. 10.1016/j.diabres.2014.04.028 24842243

[B54] Kaji K. Yoshiji H. Ikenaka Y. Noguchi R. Aihara Y. Douhara A. (2014). Dipeptidyl peptidase-4 inhibitor attenuates hepatic fibrosis via suppression of activated hepatic stellate cell in rats. *J. Gastroenterol.* 49 481–491. 10.1007/s00535-013-0783-4 23475323

[B55] Kalluri R. Zeisberg M. (2006). Fibroblasts in cancer. *Nat. Rev. Cancer* 6 392–401. 16572188 10.1038/nrc1877

[B56] Kanasaki K. Shi S. Kanasaki M. He J. Nagai T. Nakamura Y. (2014). 4 inhibition ameliorates kidney fibrosis in streptozotocin-induced diabetic mice by inhibiting endothelial-to-mesenchymal transition in a therapeutic regimen. *Diabetes* 63 2120–2131. 10.2337/db13-1029 24574044

[B57] Kang H. M. Ahn S. H. Choi P. Ko Y. A. Han S. H. Chinga F. (2015). Defective fatty acid oxidation in renal tubular epithelial cells has a key role in kidney fibrosis development. *Nat. Med.* 21 37–46. 10.1038/nm.3762 25419705 PMC4444078

[B58] Katsumata L. W. Miyajima A. Itoh T. (2017). Portal fibroblasts marked by the surface antigen Thy1 contribute to fibrosis in mouse models of cholestatic liver injury. *Hepatol. Commun.* 1 198–214. 10.1002/hep4.1023 29404454 PMC5721447

[B59] Kavian N. Servettaz A. Marut W. Nicco C. Chéreau C. Weill B. (2012). Sunitinib inhibits the phosphorylation of platelet-derived growth factor receptor β in the skin of mice with scleroderma-like features and prevents the development of the disease. *Arthrit. Rheum.* 64 1990–2000. 10.1002/art.34354 22213155

[B60] Kay J. High W. A. (2008). Imatinib mesylate treatment of nephrogenic systemic fibrosis. *Arthrit. Rheum.* 58 2543–2548. 10.1002/art.23696 18668587

[B61] Kelly T. (2005). Fibroblast activation protein-alpha and dipeptidyl peptidase IV (CD26): cell-surface proteases that activate cell signaling and are potential targets for cancer therapy. *Drug Resist. Updates* 8 51–58. 10.1016/j.drup.2005.03.002 15939342

[B62] Khalil R. Shata A. Abd El-Kader E. M. Sharaf H. Abdo W. S. Amin N. A. (2020). Vildagliptin, a DPP-4 inhibitor, attenuates carbon tetrachloride-induced liver fibrosis by targeting ERK1/2, p38α, and NF-κB signaling. *Toxicol. Appl. Pharmacol.* 407 115246. 10.1016/j.taap.2020.115246 32956689

[B63] Khanna D. Saggar R. Mayes M. D. Abtin F. Clements P. J. Maranian P. (2011). A one-year, phase I/IIa, open-label pilot trial of imatinib mesylate in the treatment of systemic sclerosis-associated active interstitial lung disease. *Arthrit. Rheum.* 63 3540–3546. 10.1002/art.30548 21769849 PMC3205223

[B64] Kim M. J. Kim N. Y. Jung Y. A. Lee S. Jung G. S. Kim J. G. (2020). Evogliptin, a Dipeptidyl Peptidase-4 inhibitor, attenuates renal fibrosis caused by unilateral ureteral obstruction in mice. *Diabetes Metab. J.* 44 186–192. 10.4093/dmj.2018.0271 31701692 PMC7043988

[B65] Kimura T. Monslow J. Klampatsa A. Leibowitz M. Sun J. Liousia M. (2019). Loss of cells expressing fibroblast activation protein has variable effects in models of TGF-β and chronic bleomycin-induced fibrosis. *Am. J. Physiol. Lung Cell. Mol. Physiol.* 317 L271–L282. 31188013 10.1152/ajplung.00071.2019

[B66] Klinkhammer B. M. Floege J. Boor P. (2018). PDGF in organ fibrosis. *Mol. Aspects Med.* 62 44–62. 10.1016/j.mam.2017.11.008 29155002

[B67] Kocabayoglu P. Lade A. Lee Y. A. Dragomir A. C. Sun X. Fiel M. I. (2015). β-PDGF receptor expressed by hepatic stellate cells regulates fibrosis in murine liver injury, but not carcinogenesis. *J. Hepatol.* 63 141–147. 10.1016/j.jhep.2015.01.036 25678385 PMC4475471

[B68] Korosec A. Frech S. Gesslbauer B. Vierhapper M. Radtke C. Petzelbauer P. (2019). Lineage identity and location within the dermis determine the function of papillary and reticular fibroblasts in human skin. *J. Invest. Dermatol.* 139 342–351. 10.1016/j.jid.2018.07.033 30179601

[B69] Kostallari E. Hirsova P. Prasnicka A. Verma V. K. Yaqoob U. Wongjarupong N. (2018). Hepatic stellate cell-derived platelet-derived growth factor receptor-alpha-enriched extracellular vesicles promote liver fibrosis in mice through SHP2. *Hepatology* 68 333–348. 10.1002/hep.29803 29360139 PMC6033667

[B70] Kuai J. Mosyak L. Brooks J. Cain M. Carven G. J. Ogawa S. (2015). Characterization of binding mode of action of a blocking anti-platelet-derived growth factor (PDGF)-B monoclonal antibody, MOR8457, reveals conformational flexibility and avidity needed for PDGF-BB to bind PDGF receptor-β. *Biochemistry* 54 1918–1929. 10.1021/bi5015425 25707433

[B71] Künzli B. M. Nuhn P. Enjyoji K. Banz Y. Smith R. N. Csizmadia E. (2008). Disordered pancreatic inflammatory responses and inhibition of fibrosis in CD39-null mice. *Gastroenterology* 134 292–305. 10.1053/j.gastro.2007.10.030 18036594 PMC2189558

[B72] Lee M. Shin E. Bae J. Cho Y. Lee J. Y. Lee Y. H. (2020). Dipeptidyl peptidase-4 inhibitor protects against non-alcoholic steatohepatitis in mice by targeting TRAIL receptor-mediated lipoapoptosis via modulating hepatic dipeptidyl peptidase-4 expression. *Sci. Rep.* 10:19429. 10.1038/s41598-020-75288-y PMC7655829 33173107

[B73] Levy M. T. McCaughan G. W. Marinos G. Gorrell M. D. (2002). Intrahepatic expression of the hepatic stellate cell marker fibroblast activation protein correlates with the degree of fibrosis in hepatitis C virus infection. *Liver* 22 93–101. 10.1034/j.1600-0676.2002.01503.x 12028401

[B74] Li Y. Zhang J. Zhou Q. Wang H. Xie S. Yang X. (2019). Linagliptin inhibits high glucose-induced transdifferentiation of hypertrophic scar-derived fibroblasts to myofibroblasts via IGF/Akt/mTOR signalling pathway. *Exp. Dermatol.* 28 19–27. 10.1111/exd.13800 30308704

[B75] Liu Y. Wang Z. Kwong S. Q. Lui E. L. H. Friedman S. L. Li F. R. (2011). Inhibition of PDGF, TGF-β, and Abl signaling and reduction of liver fibrosis by the small molecule Bcr-Abl tyrosine kinase antagonist Nilotinib. *J. Hepatol.* 55 612–625. 10.1016/j.jhep.2010.11.035 21251937

[B76] Long M. Cai L. Li W. Zhang L. Guo S. Zhang R. (2018). DPP-4 inhibitors improve diabetic wound healing via direct and indirect promotion of epithelial-mesenchymal transition and reduction of scarring. *Diabetes* 67 518–531. 29254987 10.2337/db17-0934

[B77] Lynch M. D. Watt F. M. (2018). Fibroblast heterogeneity: implications for human disease. *J. Clin. Invest.* 128 26–35. 10.1172/jci93555 29293096 PMC5749540

[B78] Mah W. Jiang G. Olver D. Gallant-Behm C. Wiebe C. Hart D. A. (2017). Elevated CD26 expression by skin fibroblasts distinguishes a profibrotic phenotype involved in scar formation compared to gingival fibroblasts. *Am. J. Pathol.* 187 1717–1735. 10.1016/j.ajpath.2017.04.017 28641076

[B79] Marcelin G. Ferreira A. Liu Y. Atlan M. Aron-Wisnewsky J. Pelloux V. (2017). A PDGFRα-mediated switch toward CD9(high) adipocyte progenitors controls obesity-induced adipose tissue fibrosis. *Cell Metab.* 25 673–685. 10.1016/j.cmet.2017.01.010 28215843

[B80] Marleau S. Harb D. Bujold K. Avallone R. Iken K. Wang Y. (2005). EP 80317, a ligand of the CD36 scavenger receptor, protects apolipoprotein E-deficient mice from developing atherosclerotic lesions. *FASEB J.* 19 1869–1871. 10.1096/fj.04-3253fje 16123174

[B81] Marques A. P. Cunha-Santos J. Leal H. Sousa-Ferreira L. Pereira de Almeida L. Cavadas C. (2018). Dipeptidyl peptidase IV (DPP-IV) inhibition prevents fibrosis in adipose tissue of obese mice. *Biochim. Biophys. Acta Gen. Subj.* 1862 403–413. 10.1016/j.bbagen.2017.11.012 29154902

[B82] Mejias M. Garcia-Pras E. Tiani C. Miquel R. Bosch J. Fernandez M. (2009). Beneficial effects of sorafenib on splanchnic, intrahepatic, and portocollateral circulations in portal hypertensive and cirrhotic rats. *Hepatology* 49 1245–1256. 10.1002/hep.22758 19137587

[B83] Nazari B. Rice L. M. Stifano G. Barron A. M. Wang Y. M. Korndorf T. (2016). Altered dermal fibroblasts in systemic sclerosis display podoplanin and CD90. *Am. J. Pathol.* 186 2650–2664. 10.1016/j.ajpath.2016.06.020 27565038 PMC5222985

[B84] Nicola T. Hagood J. S. James M. L. Macewen M. W. Williams T. A. Hewitt M. M. (2009). Loss of Thy-1 inhibits alveolar development in the newborn mouse lung. *Am. J. Physiol. Lung Cell. Mol. Physiol.* 296 L738–L750. 19270178 10.1152/ajplung.90603.2008 PMC2681351

[B85] Okamura D. M. Pennathur S. Pasichnyk K. López-Guisa J. M. Collins S. Febbraio M. (2009). CD36 regulates oxidative stress and inflammation in hypercholesterolemic CKD. *J. Am. Soc. Nephrol.* 20 495–505. 10.1681/asn.2008010009 19211715 PMC2653683

[B86] Olson L. E. Soriano P. (2009). Increased PDGFRalpha activation disrupts connective tissue development and drives systemic fibrosis. *Dev. Cell* 16 303–313. 10.1016/j.devcel.2008.12.003 19217431 PMC2664622

[B87] Ospelt C. Mertens J. C. Jüngel A. Brentano F. Maciejewska-Rodriguez H. Huber L. C. (2010). Inhibition of fibroblast activation protein and dipeptidylpeptidase 4 increases cartilage invasion by rheumatoid arthritis synovial fibroblasts. *Arthrit. Rheum.* 62 1224–1235. 10.1002/art.27395 20155839

[B88] Ostendorf T. van Roeyen C. R. Peterson J. D. Kunter U. Eitner F. Hamad A. J. (2003). A fully human monoclonal antibody (CR002) identifies PDGF-D as a novel mediator of mesangioproliferative glomerulonephritis. *J. Am. Soc. Nephrol.* 14 2237–2247. 10.1097/01.asn.0000083393.00959.02 12937299

[B89] Östman A. (2017). PDGF receptors in tumor stroma: biological effects and associations with prognosis and response to treatment. *Adv. Drug Deliv. Rev.* 121 117–123. 10.1016/j.addr.2017.09.022 28970051

[B90] Panchapakesan U. Pollock C. (2015). The role of dipeptidyl peptidase - 4 inhibitors in diabetic kidney disease. *Front. Immunol.* 6:443. 10.1042/CS20180031 26379674 PMC4551869

[B91] Papadopoulos N. Lennartsson J. The P. D. G. F. (2018). /PDGFR pathway as a drug target. *Mol. Aspects Med.* 62 75–88. 10.1016/j.mam.2017.11.007 29137923

[B92] Patel P. M. Jones V. A. Kridin K. Amber K. T. (2020). The role of Dipeptidyl Peptidase-4 in cutaneous disease. *Exp. Dermatol.* 30 304–318. 10.1111/exd.14228 33131073

[B93] Pennathur S. Pasichnyk K. Bahrami N. M. Zeng L. Febbraio M. Yamaguchi I. (2015). The macrophage phagocytic receptor CD36 promotes fibrogenic pathways on removal of apoptotic cells during chronic kidney injury. *Am. J. Pathol.* 185 2232–2245. 10.1016/j.ajpath.2015.04.016 26092500 PMC4530136

[B94] Perrot I. Michaud H. A. Giraudon-Paoli M. Augier S. Docquier A. Gros L. (2019). Blocking antibodies targeting the CD39/CD73 immunosuppressive pathway unleash immune responses in combination cancer therapies. *Cell Rep.* 27 2411.e9–2425.e9. 31116985 10.1016/j.celrep.2019.04.091

[B95] Philippeos C. Telerman S. B. Oulès B. Pisco A. O. Shaw T. J. Elgueta R. (2018). Spatial and single-cell transcriptional profiling identifies functionally distinct human dermal fibroblast subpopulations. *J. Invest. Dermatol.* 138 811–825. 10.1016/j.jid.2018.01.016 29391249 PMC5869055

[B96] Pietras K. Sjöblom T. Rubin K. Heldin C. H. Ostman A. (2003). PDGF receptors as cancer drug targets. *Cancer Cell* 3 439–443. 10.1016/s1535-6108(03)00089-8 12781361

[B97] Pontén A. Li X. Thorén P. Aase K. Sjöblom T. Ostman A. (2003). Transgenic overexpression of platelet-derived growth factor-C in the mouse heart induces cardiac fibrosis, hypertrophy, and dilated cardiomyopathy. *Am. J. Pathol.* 163 673–682. 10.1016/s0002-9440(10)63694-2 12875986 PMC1868211

[B98] Prakadan S. M. Shalek A. K. Weitz D. A. (2017). Scaling by shrinking: empowering single-cell ‘omics’ with microfluidic devices. *Nat. Rev. Genet.* 18 345–361. 10.1038/nrg.2017.15 28392571 PMC5495114

[B99] Prey S. Ezzedine K. Doussau A. Grandoulier A. S. Barcat D. Chatelus E. (2012). Imatinib mesylate in scleroderma-associated diffuse skin fibrosis: a phase II multicentre randomized double-blinded controlled trial. *Br. J. Dermatol.* 167 1138–1144. 10.1111/j.1365-2133.2012.11186.x 23039171

[B100] Rabinowitz J. D. Mutlu G. M. (2019). A metabolic strategy to reverse fibrosis? *Nat. Metab.* 1 12–13. 10.1038/s42255-018-0013-8 32694811

[B101] Ramachandran P. Dobie R. Wilson-Kanamori J. R. Dora E. F. Henderson B. E. P. Luu N. T. (2019). Resolving the fibrotic niche of human liver cirrhosis at single-cell level. *Nature* 575 512–518. 10.1038/s41586-019-1631-3 31597160 PMC6876711

[B102] Rege T. A. Hagood J. S. (2006). Thy-1 as a regulator of cell-cell and cell-matrix interactions in axon regeneration, apoptosis, adhesion, migration, cancer, and fibrosis. *FASEB J.* 20 1045–1054. 10.1096/fj.05-5460rev 16770003

[B103] Rinkevich Y. Walmsley G. G. Hu M. S. Maan Z. N. Newman A. M. Drukker M. (2015). Skin fibrosis. Identification and isolation of a dermal lineage with intrinsic fibrogenic potential. *Science* 348:aaa2151. 10.1126/science.aaa2151 25883361 PMC5088503

[B104] Roberts V. Campbell D. J. Lu B. Chia J. Cowan P. J. Dwyer K. M. (2017). The differential effect of apyrase treatment and hCD39 overexpression on chronic renal fibrosis after ischemia-reperfusion injury. *Transplantation* 101 e194–e204. 28198766 10.1097/TP.0000000000001679

[B105] Roberts V. Lu B. Chia J. Cowan P. J. Dwyer K. M. (2016). CD39 overexpression does not attenuate renal fibrosis in the unilateral ureteric obstructive model of chronic kidney disease. *Purinerg. Signal.* 12 653–660. 10.1007/s11302-016-9528-1 27565966 PMC5124005

[B106] Rothweiler S. Feldbrügge L. Jiang Z. G. Csizmadia E. Longhi M. S. Vaid K. (2019). Selective deletion of ENTPD1/CD39 in macrophages exacerbates biliary fibrosis in a mouse model of sclerosing cholangitis. *Purinerg. Signal.* 15 375–385. 10.1007/s11302-019-09664-3 31243614 PMC6737175

[B107] Sanders Y. Y. Pardo A. Selman M. Nuovo G. J. Tollefsbol T. O. Siegal G. P. (2008). Thy-1 promoter hypermethylation: a novel epigenetic pathogenic mechanism in pulmonary fibrosis. *Am. J. Respir. Cell Mol. Biol.* 39 610–618. 10.1165/rcmb.2007-0322oc 18556592 PMC2574530

[B108] Santini M. P. Malide D. Hoffman G. Pandey G. D’Escamard V. Nomura-Kitabayashi A. (2020). Tissue-resident PDGFRα(+) progenitor cells contribute to fibrosis versus healing in a context- and spatiotemporally dependent manner. *Cell Rep.* 30 555.e7–570.e7. 31940496 10.1016/j.celrep.2019.12.045 PMC7030884

[B109] Seo J. B. Choi Y. K. Woo H. I. Jung Y. A. Lee S. Lee S. (2019). Gemigliptin attenuates renal fibrosis through down-regulation of the NLRP3 inflammasome. *Diabetes Metab. J.* 43 830–839. 10.4093/dmj.2018.0181 30877711 PMC6943262

[B110] Shaikh M. V. Kala M. Nivsarkar M. (2016). CD90 a potential cancer stem cell marker and a therapeutic target. *Cancer Biomark.* 16 301–307. 10.3233/cbm-160590 27062695 PMC13016484

[B111] Shaker M. E. Salem H. A. Shiha G. E. Ibrahim T. M. (2011). Nilotinib counteracts thioacetamide-induced hepatic oxidative stress and attenuates liver fibrosis progression. *Fund. Clin. Pharmacol.* 25 248–257. 10.1111/j.1472-8206.2010.00824.x 20408881

[B112] Shaw T. J. Rognoni E. (2020). Dissecting fibroblast heterogeneity in health and fibrotic disease. *Curr. Rheumatol. Rep.* 22:33. 10.1007/s11926-020-00903-w PMC7305072 32562113

[B113] Sinha M. Sen C. K. Singh K. Das A. Ghatak S. Rhea B. (2018). Direct conversion of injury-site myeloid cells to fibroblast-like cells of granulation tissue. *Nat. Commun.* 9:936. 10.1038/s41467-018-03208-w PMC5838200 29507336

[B114] Soare A. Györfi H. A. Matei A. E. Dees C. Rauber S. Wohlfahrt T. (2020). Dipeptidylpeptidase 4 as a marker of activated fibroblasts and a potential target for the treatment of fibrosis in systemic sclerosis. *Arthrit. Rheumatol.* 72 137–149. 10.1002/art.41058 31350829

[B115] Soria A. Cario-André M. Lepreux S. Rezvani H. R. Pasquet J. M. Pain C. (2008). The effect of imatinib (Glivec) on scleroderma and normal dermal fibroblasts: a preclinical study. *Dermatology* 216 109–117. 10.1159/000111507 18216472

[B116] Souza A. C. Bocharov A. V. Baranova I. N. Vishnyakova T. G. Huang Y. G. Wilkins K. J. (2016). Antagonism of scavenger receptor CD36 by 5A peptide prevents chronic kidney disease progression in mice independent of blood pressure regulation. *Kidney Int.* 89 809–822. 10.1016/j.kint.2015.12.043 26994575 PMC4800337

[B117] Spiera R. F. Gordon J. K. Mersten J. N. Magro C. M. Mehta M. Wildman H. F. (2011). Imatinib mesylate (Gleevec) in the treatment of diffuse cutaneous systemic sclerosis: results of a 1-year, phase IIa, single-arm, open-label clinical trial. *Ann. Rheum. Dis.* 70 1003–1009. 10.1136/ard.2010.143974 21398330 PMC3086082

[B118] Suwanai H. Watanabe R. Sato M. Odawara M. Matsumura H. (2020). Dipeptidyl peptidase-4 inhibitor reduces the risk of developing hypertrophic scars and keloids following median sternotomy in diabetic patients: a nationwide retrospective cohort study using the national database of health insurance claims of Japan. *Plast. Reconstruct. Surg.* 146 83–89. 10.1097/prs.0000000000006904 32590649

[B119] Suzuki T. Tada Y. Gladson S. Nishimura R. Shimomura I. Karasawa S. (2017). Vildagliptin ameliorates pulmonary fibrosis in lipopolysaccharide-induced lung injury by inhibiting endothelial-to-mesenchymal transition. *Respir. Res.* 18:177. 10.1186/s12931-017-0660-4 PMC5644255 29037205

[B120] Sweetwyne M. T. Murphy-Ullrich J. E. (2012). Thrombospondin1 in tissue repair and fibrosis: TGF-β-dependent and independent mechanisms. *Matrix Biol.* 31 178–186. 10.1016/j.matbio.2012.01.006 22266026 PMC3295861

[B121] Tabib T. Morse C. Wang T. Chen W. Lafyatis R. (2018). SFRP2/DPP4 and FMO1/LSP1 define major fibroblast populations in human skin. *J. Invest. Dermatol.* 138 802–810. 10.1016/j.jid.2017.09.045 29080679 PMC7444611

[B122] Takagaki Y. Koya D. Kanasaki K. (2017). Dipeptidyl peptidase-4 inhibition and renoprotection: the role of antifibrotic effects. *Curr. Opin. Nephrol. Hypertens.* 26 56–66. 10.1097/mnh.0000000000000291 27820706

[B123] Tan C. Jiang M. Wong S. S. Espinoza C. R. Kim C. Li X. (2019). Soluble Thy-1 reverses lung fibrosis via its integrin-binding motif. *JCI insight* 4:e131152. 10.1172/jci.insight.131152 PMC6948761 31672942

[B124] Thielitz A. Vetter R. W. Schultze B. Wrenger S. Simeoni L. Ansorge S. (2008). Inhibitors of dipeptidyl peptidase IV-like activity mediate antifibrotic effects in normal and keloid-derived skin fibroblasts. *J. Invest. Dermatol.* 128 855–866. 10.1038/sj.jid.5701104 17943180

[B125] Tillmanns J. Hoffmann D. Habbaba Y. Schmitto J. D. Sedding D. Fraccarollo D. (2015). Fibroblast activation protein alpha expression identifies activated fibroblasts after myocardial infarction. *J. Mol. Cell. Cardiol.* 87 194–203. 10.1016/j.yjmcc.2015.08.016 26319660

[B126] Truffi M. Sorrentino L. Monieri M. Fociani P. Mazzucchelli S. Bonzini M. (2018). Inhibition of fibroblast activation protein restores a balanced extracellular matrix and reduces fibrosis in crohn’s disease strictures ex vivo. *Inflamm. Bowel Dis.* 24 332–345. 10.1093/ibd/izx008 29361086

[B127] Uchii M. Kimoto N. Sakai M. Kitayama T. Kunori S. (2016). Glucose-independent renoprotective mechanisms of the tissue dipeptidyl peptidase-4 inhibitor, saxagliptin, in Dahl salt-sensitive hypertensive rats. *Eur. J. Pharmacol.* 783 56–63. 10.1016/j.ejphar.2016.04.005 27063445

[B128] Vickovic S. Eraslan G. Salmén F. Klughammer J. Stenbeck L. Schapiro D. (2019). High-definition spatial transcriptomics for in situ tissue profiling. *Nat. Methods* 16 987–990. 10.1038/s41592-019-0548-y 31501547 PMC6765407

[B129] Vorstandlechner V. Laggner M. Kalinina P. Haslik W. Radtke C. Shaw L. (2020). Deciphering the functional heterogeneity of skin fibroblasts using single-cell RNA sequencing. *FASEB J.* 34 3677–3692. 10.1096/fj.201902001rr 31930613

[B130] Vuorinen K. Gao F. Oury T. D. Kinnula V. L. Myllärniemi M. (2007). Imatinib mesylate inhibits fibrogenesis in asbestos-induced interstitial pneumonia. *Exp. Lung Res.* 33 357–373. 10.1080/01902140701634827 17849262 PMC2652685

[B131] Wang X. Chen Y. Lv L. Chen J. (2009). Silencing CD36 gene expression results in the inhibition of latent-TGF-beta1 activation and suppression of silica-induced lung fibrosis in the rat. *Respir. Res.* 10:36. 10.1186/1465-9921-10-36 PMC2698900 19439069

[B132] Wang X. M. Yu D. M. McCaughan G. W. Gorrell M. D. (2005). Fibroblast activation protein increases apoptosis, cell adhesion, and migration by the LX-2 human stellate cell line. *Hepatology* 42 935–945. 10.1002/hep.20853 16175601

[B133] Wang Y. M. McRae J. L. Robson S. C. Cowan P. J. Zhang G. Y. Hu M. (2012). cells participate in CD39-mediated protection from renal injury. *Eur. J. Immunol.* 42 2441–2451. 10.1002/eji.201242434 22684996

[B134] Wei K. Korsunsky I. Marshall J. L. Gao A. Watts G. F. M. Major T. (2020). Notch signalling drives synovial fibroblast identity and arthritis pathology. *Nature* 582 259–264. 10.1038/s41586-020-2222-z 32499639 PMC7841716

[B135] Worthen C. A. Cui Y. Orringer J. S. Johnson T. M. Voorhees J. J. Fisher G. J. (2020). CD26 identifies a subpopulation of fibroblasts that produce the majority of collagen during wound healing in human skin. *J. Invest. Dermatol.* 140 2515.e3–2524.e3. 32407715 10.1016/j.jid.2020.04.010 PMC7655599

[B136] Xin Y. Wang X. Zhu M. Qu M. Bogari M. Lin L. (2017). Expansion of CD26 positive fibroblast population promotes keloid progression. *Exp. Cell Res.* 356 104–113. 28454879 10.1016/j.yexcr.2017.04.021

[B137] Xu J. Wang J. He M. Han H. Xie W. Wang H. (2018). Dipeptidyl peptidase IV (DPP-4) inhibition alleviates pulmonary arterial remodeling in experimental pulmonary hypertension. *Lab. Invest.* 98 1333–1346. 10.1038/s41374-018-0080-1 29789684

[B138] Yang R. Elsaadi S. Misund K. Abdollahi P. Vandsemb E. N. Moen S. H. (2020). Conversion of ATP to adenosine by CD39 and CD73 in multiple myeloma can be successfully targeted together with adenosine receptor A2A blockade. *J. Immunother. Cancer* 8:e000610. 10.1136/jitc-2020-000610 32409420 PMC7239696

[B139] Yang X. Okamura D. M. Lu X. Chen Y. Moorhead J. Varghese Z. (2017). CD36 in chronic kidney disease: novel insights and therapeutic opportunities. *Nat. Rev. Nephrol.* 13 769–781. 10.1038/nrneph.2017.126 28919632

[B140] Yang Y. L. Lin S. H. Chuang L. Y. Guh J. Y. Liao T. N. Lee T. C. (2007). CD36 is a novel and potential anti-fibrogenic target in albumin-induced renal proximal tubule fibrosis. *J. Cell. Biochem.* 101 735–744. 10.1002/jcb.21236 17226761

[B141] Yehualaeshet T. O’Connor R. Begleiter A. Murphy-Ullrich J. E. Silverstein R. Khalil N. (2000). A CD36 synthetic peptide inhibits bleomycin-induced pulmonary inflammation and connective tissue synthesis in the rat. *Am. J. Respir. Cell Mol. Biol.* 23 204–212. 10.1165/ajrcmb.23.2.4089 10919987

[B142] Ying H. Z. Chen Q. Zhang W. Y. Zhang H. H. Ma Y. Zhang S. Z. (2017). PDGF signaling pathway in hepatic fibrosis pathogenesis and therapeutics (Review). *Mol. Med. Rep.* 16 7879–7889. 10.3892/mmr.2017.7641 28983598 PMC5779870

[B143] Yoshida S. Ikenaga N. Liu S. B. Peng Z. W. Chung J. Sverdlov D. Y. (2014). Extrahepatic platelet-derived growth factor-β, delivered by platelets, promotes activation of hepatic stellate cells and biliary fibrosis in mice. *Gastroenterology* 147 1378–1392. 10.1053/j.gastro.2014.08.038 25173753

[B144] Zhang H. Sun D. Wang G. Cui S. Field R. A. Li J. (2019). Alogliptin alleviates liver fibrosis via suppression of activated hepatic stellate cell. *Biochem. Biophys. Res. Commun.* 511 387–393. 10.1016/j.bbrc.2019.02.065 30797555

[B145] Zhao X. Psarianos P. Ghoraie L. S. Yip K. Goldstein D. Gilbert R. (2019). Metabolic regulation of dermal fibroblasts contributes to skin extracellular matrix homeostasis and fibrosis. *Nat. Metab.* 1 147–157. 10.1038/s42255-018-0008-5 32694814

